# Analysing Time-Stamped Co-Editing Networks in Software Development Teams using git2net

**DOI:** 10.1007/s10664-020-09928-2

**Published:** 2021-05-26

**Authors:** Christoph Gote, Ingo Scholtes, Frank Schweitzer

**Affiliations:** 1 Chair of Systems Design, ETH Zurich, Zurich, Switzerland; 2 Data Analytics Group, Bergische Universität Wuppertal, Wuppertal, Germany

**Keywords:** Repository mining, Collaboration networks, Developer productivity

## Abstract

Data from software repositories have become an important foundation for the empirical study of software engineering processes. A recurring theme in the repository mining literature is the inference of developer networks capturing e.g. collaboration, coordination, or communication from the commit history of projects. Many works in this area studied networks of *co-authorship* of software artefacts, neglecting detailed information on code changes and code ownership available in software repositories. To address this issue, we introduce git2net, a scalable python software that facilitates the extraction of fine-grained *co-editing networks* in large git repositories. It uses text mining techniques to analyse the detailed history of textual modifications *within* files. We apply our tool in two case studies using GitHub repositories of multiple Open Source as well as a proprietary software project. Specifically, we use data on more than 1.2 million commits and more than 25,000 developers to test a hypothesis on the relation between developer productivity and co-editing patterns in software teams. We argue that git2net opens up an important new source of high-resolution data on human collaboration patterns that can be used to advance theory in empirical software engineering, computational social science, and organisational studies.

**Supplementary Information:**

The online version contains supplementary material available at (10.1007/s10664-020-09928-2).

## Introduction

Software repositories are a rich source of data facilitating empirical studies of software engineering processes. Methods to use meta-data from these repositories have become a common theme in the repository mining literature. Thanks to the availability of massive databases, it has become easy to query meta-data on the commits of developers at large scale (Gousios and Spinellis 2012; 2017). Apart from the evolution of software artefacts, such data also contain a wealth of fine-grained information on the human and social aspects of software development teams. Specifically, the commit history of developers allows to construct social networks that proxy collaboration, coordination, or communication structures in software teams. These databases have therefore facilitated data-driven studies of social systems not only in empirical software engineering, but also in areas like computational social science, social network analysis, organisational theory, or management science (Carley and Wallace 2001; Von Krogh and Von Hippel 2006).

The detailed record of file modifications contained in the commit log of, e.g. git repositories also enables more advanced network reconstruction techniques. In particular, from the micro-level analysis of textual modifications between subsequent versions of code we can infer *time-stamped, weighted, and directed co-editing relationships*. Such a relationship (*A*, *B*;*t*, *w*) indicates that at time *t* developer *A* modified *w* characters of code originally written by another developer *B*. Recent research has shown that such a fine-grained analysis of co-editing networks in large software projects can provide insights that go beyond more coarse-grained definitions (Joblin et al. 2015; Scholtes et al. 2016). However, a tool to conveniently extract such rich, time-stamped collaboration networks for the large corpus of git repositories available, e.g. via public platforms like GitHub, is currently missing.

Addressing this gap, we first introduce git2net, a tool that facilitates the scalable extraction of time-stamped co-editing relationships between developers in large software repositories. We then apply git2net in a large-scale analysis of coordination overhead in software development teams.

The contributions of our work are as follows:

- We introduce git2net, a python tool that can be used to mine time-stamped co-editing relations between developers from the sequence of file modifications contained in git repositories. Building on the repository mining framework pyDriller (Spadini et al. 2018), git2net can operate both on local and remote repositories. Providing a command-line interface as well as an API, git2net can be used as a stand-alone tool for standard analysis tasks and as a framework for the implementation of advanced data mining scripts. Our tool is available as an Open Source project^1^.
- Analysing all file modifications contained in the commit log, git2net generates a database that captures fine-grained information on co-edited code either at the level of lines or contiguous code regions. Building on text mining techniques, it further analyses the overlap between co-edited code regions using (i) the Levenshtein edit distance (Levenshtein 1966) and (ii) a text-based entropy measure (Shannon 1948). These measures facilitate (a) a character-based proxy estimating the effort behind code modifications, and (b) an entropy-based correction for binary file changes that can have a considerable impact on text-based and commit-based development effort estimation techniques.
- We develop an approach to generate time-stamped collaboration networks based on multiple projections: (i) time-stamped co-editing networks, (ii) time-stamped bipartite networks linking developers to edited files, and (iii) directed acyclic graphs of code edits that allow to infer “paths” of consecutive edits building upon each other. All network projections are implemented in git2net and can be directly exported as HTML visualisations as well as formats readable by common network analysis tools including pathpy (Scholtes 2017a), igraph (Csardi and Nepusz 2006), NetworkX (Hagberg et al. 2008), graph-tool (Peixoto 2014), and Gephi (Bastian et al. 2009).
- Thanks to a parallel processing model that utilises modern multi-core architectures, git2net supports the analysis of massive software repositories with hundreds of thousands of commits and millions of lines of code. A scalability analysis proves that our parallel implementation yields a linear speed-up compared to a single-threaded implementation, thus facilitating the fine-grained textual analysis even in massive projects with a long history.
- Utilising git2net in a case study on two software projects, we show that the fine-grained textual analysis of file modifications yields considerably different network structures compared to coarse-grained methods that analyse code co-authorship at the level of files or modules.
- We finally demonstrate how our tool can be used to segment developer effort into (a) the revision of code authored by the developer him- or herself vs. (b) the revision of code written by other team members. Using data on six large Open Source software projects, we take a microscopic view on coordination in software development teams. Our findings substantiate the hypothesis that the overhead of coordination is a key mechanism that drives the Ringelmann effect in collaborative software development (Scholtes et al. 2016).

This article is an extended version of the MSR 2019 contribution “git2net – Mining Time-Stamped Co-Editing Networks from Large git Repositories” Gote et al. (2019b). Providing a novel method to mine fine-grained collaboration networks at high temporal resolution from any git repository, our work opens new perspective for empirical studies of development processes. It further contributes a simple method to generate data on temporal social networks that are of interest for researchers in computational social science, (social) network analysis and organisational theory. In this version of our work we have extended git2net to facilitate the extraction of changes and co-edits from merge commits. We further include a new method to infer line-editing paths representing successive edits made to individual lines. Finally, we add a large-scale analysis in which we study coordination overhead in six collaborative Open Source Software projects.

In the following, we outline the structure of the remainder of this paper. In Section 2, we discuss relevant related work. Section 3 introduces our proposed methodology to extract time-resolved and directed links between developers who subsequently edit each others’ code. Section 4 presents a case study, in which we apply our tool to git repositories from (i) an Open Source Software project, and (ii) a proprietary, closed-source project. In Section 5, we present an empirical study of factors that influence the productivity of developers in collaborative software projects. Section 6 highlights the next steps in our research, and outlines research questions and hypotheses that can be addressed and tested using git2net. The threats to validity of our work are discussed in Section 7. Finally, in Section 8 we draw conclusions from our work.

## Related Work

Considering the two-fold contributions of our work, namely providing a tool to extract fine-grained developer co-editing networks and using this tool to empirically study coordination overhead in software teams, we split the discussion of related work in two sections. In the first section, we review related works that have applied social network analysis to software development teams, while in the second section we review works studying how coordination in software teams influences the productivity of developers.

### Constructing Social Networks from Software Repositories

Given the large body of work using network analysis to study software development processes, we restrict our overview to related works that address the reconstruction of social networks from software repositories. A broader view on applications of graph-based data analysis and modelling techniques in empirical software engineering—including works on (technical) dependency networks that are outside the scope of our work—is, e.g., available in Wolf et al. (2009b), Xie et al. (2009), and Cataldo et al. (2014).

A number of studies use operational data on software projects to construct graphs or networks where nodes capture developers while links capture social interactions and/or work dependencies between developers. To this end, a first line of works has used data that directly capture communication (Geipel et al. 2014), e.g. via IRC channels (Cataldo and Herbsleb 2008), E-Mail exchanges (Bird et al. 2006; Wolf et al. 2009a; Bacchelli et al. 2011; Hong et al. 2011; Xuan and Filkov 2014), mailing lists (Guzzi et al. 2013), or communication via issue trackers (Long and Siau 2007; Howison et al. 2006; Sureka et al. 2011), Zanetti et al. (2013a, b).

While data on direct developer communication facilitate the construction of meaningful social networks, they are often not available, e.g. due to privacy concerns. To address such settings, researchers have developed methods to *infer* or *reconstruct* collaboration networks based on developer actions recorded in code repositories like CVS, SVN, or git. A common approach starts from *code authorship* or *code ownership* networks, which map the relation between a developer and the artefacts (i.e. files, modules, binaries, etc.) that he or she contributed to Fritz et al. (2007), Bird et al. (2011), Greiler et al. (2015), MacLean and Knutson (2013), and Posnett et al. (2013). The resulting directed bipartite developer-artefact networks can then be projected onto *co-authorship networks*, where undirected links between two developers *A* and *B* indicate that *A* and *B* have modified at least one common artefact. Geipel and Schweitzer (2009) and Geipel (2012) have studied co-change based on a large corpus of CVS repositories of Open Source Software projects.

The majority of works mining social networks from software repositories build on this general idea of co-authorship networks. In MacLean and Knutson (2013), Madey et al. (2002), Meneely et al. (2008), Ogawa and Ma (2010), and Vijayaraghavan et al. (2015) a file-based notion of co-authorship is used to construct *co-commit networks*, where a link between two developers signifies that they have committed the same file at least once. Lopez-Fernandez et al. (2004) adopt a module-based definition, assuming that two developers are linked in the co-authorship network if they have contributed to at least one common module. Taking a similar approach, Huang and Liu (2005) use information on modified file paths in SourceForge repositories to infer relations between authors editing the same part of a project. Incorporating the time stamps of commits, Pohl and Diehl (2008) used a file-based co-authorship definition to construct *dynamic* developer networks that can be analysed and visualised using methods from dynamic network analysis (Holme 2015). Cohen and Consens (2018) recently developed a similar approach to study the ecosystem of software projects on GitHub. To this end, they define project-level co-commit networks, i.e. a projection of commits where two developers are linked if they committed to the same Open Source project. Schweitzer et al. (2014) provided a related study, analysing ten years of data from the Open Source project hosting platform SourceForge.

These works have typically constructed *undirected co-authorship networks* based on joint contributions to files, modules, or projects. Such coarse-grained definitions of co-authorship networks introduce a potential issue: They do not distinguish between (i) links between developers that are due to *independent* contributions to the same artefact, and (ii) links that are due to commit sequences where one developer builds upon and/or redacts the particular lines of source code previously authored by another developer. Networks defined based on the latter type of *time-ordered co-editing* of code regions are likely associated with a stronger need for coordination and communication than the mere fact that developers edited the same file or module (Cataldo et al. 2006). So far, few studies have adopted such fine-grained approaches to create developer collaboration networks. Notable exceptions include the function-level co-editing networks constructed by Joblin et al. (2015). The authors further argue that, using file-based definitions of collaboration networks, network analytic methods fail to identify meaningful communities. Scholtes et al. (2016) constructed line-based co-editing networks, showing that such an analysis (i) yields insights into the coordination structures of software teams, and (ii) provides new ways to test long-standing hypotheses about cooperative work from social psychology.

While such a fine-grained analysis of the co-editing behaviour of developers has its advantages, it also introduces challenges that have so far limited its adoption. First and foremost, it requires a detailed analysis of file modifications and makes it necessary to identify the original author for every modified line of code affected in each commit. Requiring a potentially large number of git operations for every commit being analysed, such an analysis is both complicated to implement as well as time-consuming to perform. Compared to other approaches, which often merely require a suitable query in structured databases like ghTorrent (Gousios and Spinellis^2012^, ^2017^), a tool that facilitates this task for very large repositories is still missing.

Closing this gap, our work introduces a practical and scalable solution for the construction of fine-grained and time-stamped co-editing networks from git repositories. Our work extends the state-of-the-art and facilitates analyses of developer collaboration and coordination in software projects. Providing a new method to construct large, dynamic networks at high temporal resolution we further expect our work to be of interest for the community of researchers developing methods to analyse dynamic (social) networks (Holme 2015; Berger-Wolf and Saia 2006; Carley and Pfeffer 2012).

### Relation Between Individual Productivity and Team Size

A key motivation behind the network-based study of collaborative software development is to improve our understanding of how social aspects in software teams influence the efficiency, performance, or success of projects. One line of research in this area has focused on the question how the size and structure of software teams affects the individual productivity of developers. While some works argue that a growing team size is associated with an increase of individual productivity (Sornette et al. 2014; Muric et al. 2019), other studies have come to the opposite conclusion (Brooks FP 1975; Scholtes et al. 2016; Adams et al. 2009; Paiva et al. 2010). The latter finding can be explained by the so-called Ringelmann effect, i.e. the finding that the individual productivity of co-workers in a group tends to decrease as the group grows larger (Ringelmann 1913). The Ringelmann effect was originally discovered in a 19th century human experiment that is now frequently cited as one of the classical experiments in social psychology (Kravitz and Martin 1986). Studying the effect in collaborative software development, a recent work by Scholtes et al. (2016) has tested the hypothesis that the productivity of individual developers is negatively correlated with group size. Through a large-scale study analysing the git repositories of 58 major Open Source software projects with more than 30,000 developers and more than 500,000 commits, this work has presented evidence for a strong Ringelmann effect. At the same time, it provides a quantitative underpinning for *Brooks’ law of software project management*, which is often paraphrased as “adding manpower to a late software project makes it later” Brooks FP (1975).

A number of potential mechanisms that could explain a decrease in productivity as software teams grow in size have been proposed in social psychology, organisational theory, and empirical software engineering. An exhaustive review of this research is beyond the scope of our article, therefore we refer the interested reader to the summary of related works in Scholtes et al. (2016). Here, we limit our discussion to two dominant explanations for the effect, namely (i) psychological factors that negatively impact the motivation of individuals in a group (Latané et al. 1979), and (ii) the growing overhead associated with the coordination of work between contributors as teams grow in size. Investigating the latter mechanism, Scholtes et al. (2016) have employed a *macroscopic* study of co-editing networks in major Open Source software projects. The key idea behind this analysis is that the *density* of co-editing networks—extracted from the developers’ commit log as explained in Section 3.2—yields a proxy for the coordination overhead that results from the pattern in which developers are required to edit code previously written by other team members. The results show that the strength of the Ringelmann effect is statistically related to the “densification” of co-editing networks as teams grow in size (Scholtes et al. 2016). In particular, co-editing networks that are more densely connected correspond to situations where developers need to coordinate their changes with a larger number of other developers (Scholtes et al. 2016).

Extending this related work, our present study shows how git2net can be used to advance our understanding of social mechanisms that are likely to drive the Ringelmann effect in collaborative software engineering. We particularly use the co-editing networks constructed by git2net to assess how the amount of edited code originally written by other developers influences the amount of code produced within a given time. Our analysis is based on the hypothesis that the frequent editing of code written by other developers entails a larger coordination overhead (Blincoe et al. 2013), which is likely to negatively influence the amount of contributed code.

## Mining Co-Editing Relations from git Repositories

### From Commit Logs to Co-Edits

We first outline our proposed method to extract co-editing relationships from git commits. An overview of the mining procedure, which we will explain in the following, is presented in Algorithm 1.

git projects generally consist of multiple files that can be edited by a large number of developers. Sets of changes made by a developer to potentially multiple files are recorded as commits, where each commit is identified by a unique hash. Building on the package pydriller (Spadini et al. 2018), we first extract the history of all commits in a repository and record the meta-data (author, time of commit, branch, etc.) for each commit. As the person committing the changes is not necessarily the author of these changes (a different developer can commit code on behalf of the original author), both the committer and author of the changes are considered. Subsequently we analyse the changes made with the commit.

As each commit can contain modifications of multiple files, we analyse each file modification individually to associate every changed text region with its original author. In a first step, we select the modifications relevant for the current analysis. To this end, we have implemented a filter allowing to exclude specific files, file types as well as entire directories or sub-directories from the analysis. For all selected modifications, the associated diff is analysed, determining which lines have been added or deleted. In addition, we identify the original author of every edited line of code by executing git blame on the version of the analysed file before the current commit. git blame is a very versatile tool that annotates all lines of a file with the commit that last modified them. To this end, it provides users a number of options that change its behaviour to different settings. Two of these options are -C and -w that can be specified within the options of git2net and are passed on to git blame. The -C option allows the detection of lines moved or copied between files in the same commit. The option can be provided two or three times to also detect copies from files in the commit originally creating the file or any previous commit, respectively. As using the -C is computationally expensive it is disabled by default. Aiming for a compromise between accuracy and computational complexity, all repositories shown in this work mined with -C activated once. Setting the -w options causes git blame to ignore whitespaces during line comparisons. Hence, by using it, edits that only change whitespaces can be ignored. For this reason, the option was used for all repositories discussed in this work. However, also the -w is disabled by default to obtain results identical to the ones obtained when using git blame on GitHub. Apart from the user specified options for -C and -w, we always use the option –show-number to cause git blame to identify and show the corresponding line number in the original commit. Finally, we use –line-porcelain to facilitate processing of the returned blame data.

By matching the author *A* of a modification contained in the current commit with time stamp *t* to the authors *B*_*i*_ of the previous modification of an edited line *i*, we obtain time-stamped and directed co-editing relations (*A*, *B*_*i*_,*t*). 
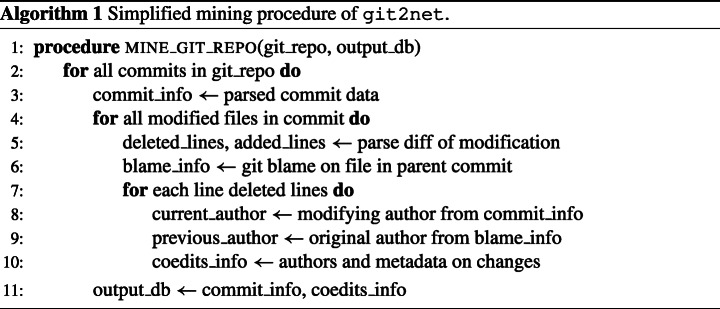


For each extracted relation, we record hashes of the original and modifying commit as well as meta-data capturing the location (file name, line number) of the associated co-edit. Naturally, such co-edits can be linked to vastly different development effort, ranging from a change of whitespaces to the complete rewriting of code. To capture to what extent developers edit each others’ code, we use a text mining approach to address these differences. We specifically use the Levenshtein edit distance (Levenshtein 1966), which can be thought of as the minimum number of keystrokes required to transform the prior source code version into the version after the edit. This measure proxies the development effort associated with an edit, where single character changes, line deletions, or the commenting/uncommenting of lines are associated with a minimum effort while the writing of a new line of code is associated with maximum effort. This approach allows us to construct time-stamped and *weighted* co-edit relations (*A*, *B*;*t*, *w*), where the weight *w* captures the Levenshtein distance of the associated edit.

An issue that we have encountered during the testing of our method in real-world repositories is associated with the embedding of text-encoded binary objects in source code, e.g. due to the inclusion of base64-encoded images in HTML or JavaScript. Notably, the modification of a single pixel in a text-encoded image, can result in a completely different text encoding. Considering our approach to associate the weight of a co-edit relation with the Levensthein edit distance this can considerably distort our analysis, potentially leading to the issue that binary file modifications dominate the recorded weights. We take an information-theoretic approach to enable the detection (and potential exclusion) of such modifications. In particular, we compute the entropy *S* of code before and after the change, defined as:

1 $$ \begin{array}{@{}rcl@{}} S = -\underset{k}{\sum} \textbf{p}_{k} \log_{2}(\textbf{p}_{k}) \end{array} $$

**Fig. 1:**
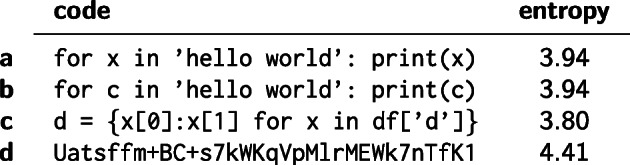
Entropy of equal length strings based on discrete utf-8 (256 possible symbols) probability space. The entropy can take values between 0 and 8 bits. The entropy of base64 encoded image (**d**) is considerably higher than of typical lines of (python) code (**a**–**c**). In practice the effect is amplified as strings of binary encoded images are longer. Small changes within a line have a small or no effect on entropy as can be seen in the entropy difference between **a** and **b**

This computation is based on the utf-8 encoding space with 256 possible symbols. Entries of the vector **p** represent a symbol’s normalised frequency in a given string. Given this definition, the entropy *S* can take values between 0 and 8 bits. Some examples for this measure are given in Fig. 1. The resulting distribution of entropy for all co-edits can be used for a Bayesian classification distinguishing, e.g. binary encoded images or hashes from natural language or source code changes.

**Fig. 2:**
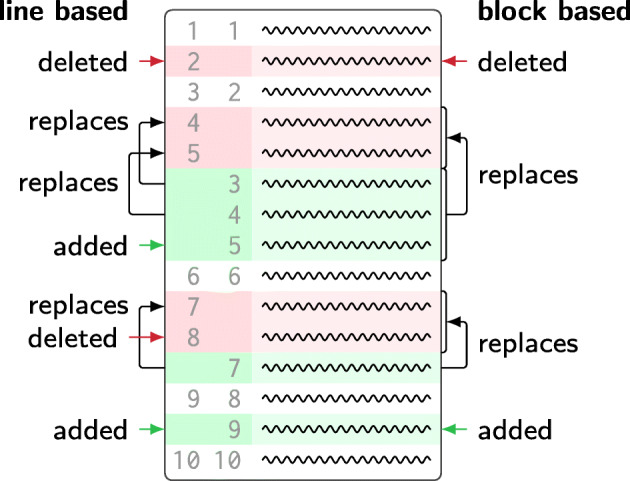
Identification of replacements using line- and block-based analysis

In the discussion above, we have considered a purely line-based approach, which treats every modified line of code as a separate entity. However, it is common that developers edit contiguous regions of code, consisting of multiple adjacent lines, with a single modification. As illustrated in Fig. 2, git2net therefore provides an option to analyse co-edits at the granularity of such contiguous code regions rather than lines. Compared to previous approaches, which have used programming language constructs like functions to identify co-edits at a granularity smaller than files (Joblin et al. 2015), this approach has the advantage that it is agnostic of the programming language. It further allows to analyse co-edit relations in files that do not represent source code, e.g. in text documents.

To explain our approach of identifying edited *blocks* of code, we distinguished between different cases contained in Fig. 2: For deleted lines (e.g. line 2 in Fig. 2) a normal co-editing relationship is recorded. Edits exclusively consisting of added lines are recorded in the database but not considered as co-edits (neither by a line-based nor by a block-based approach) as no previous author exists. The Levenshtein edit distance for pure additions matches the number of characters that were added. For cases where a set *D* of deleted lines is replaced by a set *A* of added lines, the line-based approach matches each line *d*_*i*_ ∈ *D* with a line *a*_*i*_ ∈ *A* for $i \leq \min \limits (\vert D \vert , \vert A \vert )$. If |*D*| < |*A*|, a line-based approach would thus treat the excess lines in *A* as added lines, thus not considering them as a co-edit. This is the case in line 4-5 in Fig. 2. With our block-based approach, we instead identify that a block of lines (lines 4-5) in the original file is replaced by a new block (lines 3-5) in the new file. If |*D*| > |*A*|, a line-based approach identifies the excess lines in *D* as deleted lines (see line 7-8 in Fig. 2). Through a block-based analysis we are instead able to identify that a block of lines (lines 7-8) in the original file is replaced by a new block of lines (line 7) in the new file.

While for the line-based approach, all editing statistics such as the Levenshtein edit distance or the entropy are computed on pairs of lines (*d*_*i*_,*a*_*i*_), the block based approach considers the set of lines in *A* as a replacement of the lines contained in *D*. Consequently all statistics are computed for the pair of code blocks (*D*, *A*).

**Fig. 3:**
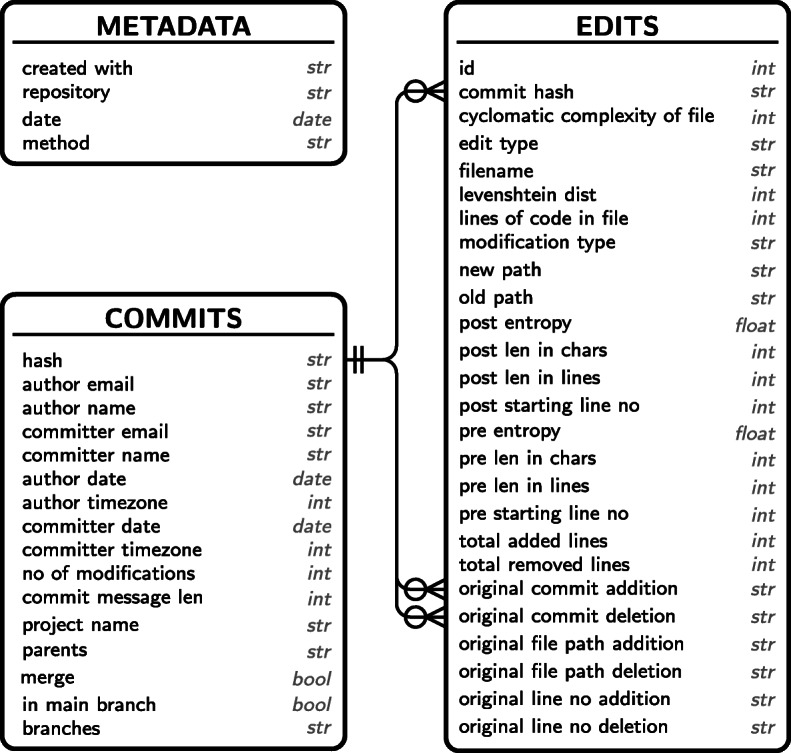
Relations in the co-editing database

After evaluating each commit, results are written to an sqlite database. This allows to pause and resume an analysis at any point in time and helps to prevent data loss from system crashes. The resulting database scheme is shown in Fig. 3.

### From Co-Edits to Networks

Given the database of co-editing relationships generated by the approach described above, git2net provides procedures to generate three different types of network projections: (i) co-editing networks, (ii) directed acyclic graphs of edit sequences for a given file, and (iii) bipartite networks linking developers to edited files.

**Fig. 4:**
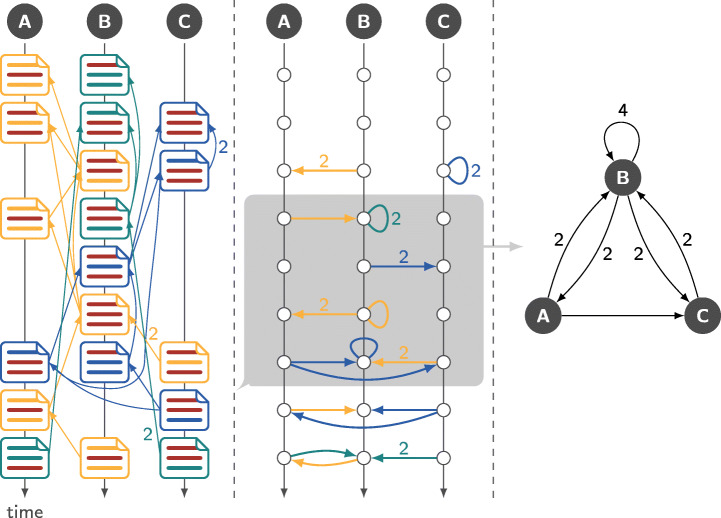
Process of generating a co-editing network from git commits. To enhance readability, each commits only modifies a single file. Three different colour coded files are considered. Edited lines are shown in red. For all edits, edges to the commit containing the original line are shown on the left hand side. Link weights are determined based on the number of lines changed. A time stamped link between the authors of the modified lines is recorded once the edit takes place (cf. centre figure). The resulting set of time stamped edges can either be analysed itself or aggregated into co-editing networks via a sliding window analysis as shown on the right. Unless indicated otherwise, all edge weights are 1

The process of generating co-editing networks is illustrated in an example shown in Fig. 4. The left column shows three developers (*A*, *B*, and *C*) editing three colour-coded files. Modified lines are shown in red. Edges between files represent the number of overlapping lines, which for illustrative purposes we show instead of the more granular Levenshtein edit distance. Given these edges, we generate a temporal network connecting the developers (cf. Fig. 4, centre for a time-unfolded representation). A link (*A*, *B*;*t*, *w*) in this network represents a commit by developer *A* at time *t* in which *w* lines originally authored by developer *B* are modified. By the aggregation of time-stamped links over a (moving) time window we obtain co-editing networks as shown in the right column of Fig 4.

Apart from co-editing networks, git2net supports the construction of file-based directed acyclic graphs (DAGs) of commits based on co-editing relationships.

**Fig. 5:**
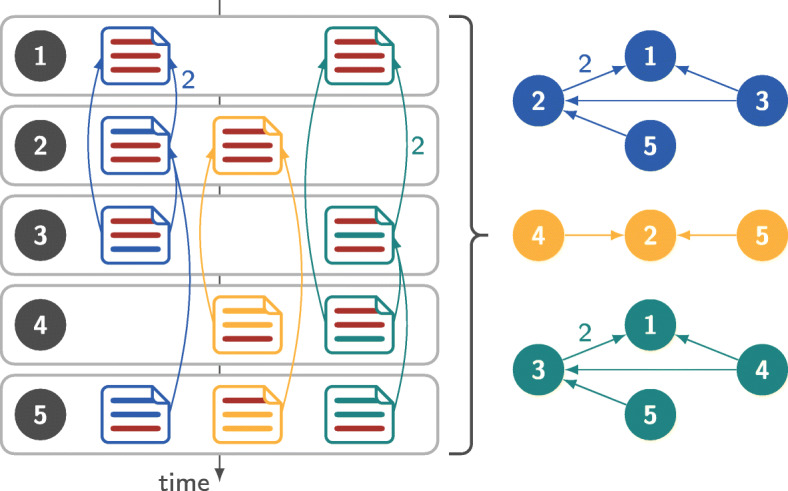
Process of creating file based directed acyclic co-editing graphs. The left hand side shows a set of commits modifying three colour coded files. For each file a directed acyclic graph is generated linking consecutive commits with overlapping changes

Each path in this DAG represent a sequence of consecutive co-editing relationships of developers editing the given file, i.e. a sequence of commits containing file modifications that built upon each other. The nodes in this graph represent commits and edges represent co-editing relationships between the authors of the commits. An example for the construction of such a DAG from a set of five commits containing file modifications is shown in Fig. 5. Individual connected components of the DAG represent proxies of knowledge flow for this file. This has been highly valuable in our own research as it immediately allows the extraction of paths from the co-editing relationships. Analysing these paths with the methods provided by the software package pathpy (Scholtes 2017a) allows to trace knowledge flow within specific areas of the development—a topic we identified as highly relevant in discussions with practitioners from software development companies.

To additionally facilitate coarse-grained analyses at the level of file-based co-authorship relations, git2net finally supports the construction of bipartite file-developer networks, where directed links (*d*, *f*) ∈ *D* × *F* indicate that a developer *d* ∈ *D* has modified a file *f* ∈ *F*.

### Line-Editing Paths

Editing a line of code in a project typically requires effort to coordinate with the previous author of the line. This can range from reminding oneself of the functionality of a line—e.g. when editing own code—or trying to understand the underlying rationale of code authored by other developers. We can thus interpret a co-edit as a flow of information between developers. Previous literature has often made use of co-authorship networks to study this information exchange (MacLean and Knutson 2013; Madey et al. 2002; Meneely et al. 2008; Ogawa and Ma 2010; Vijayaraghavan et al. 2015). Through the introduction of co-editing networks, where two developers are connected if they edited the same line, git2net increases the granularity of such analyses to the level of the individual characters.

In (Scholtes et al. 2014), the authors showed that not only the existence of interactions between developers matters, but that the order in which these interactions occur can have a crucial effect on dynamical processes such as knowledge diffusion. To facilitate the study of consecutive changes to lines git2net is capable of extracting time-ordered line-editing paths from git repositories.

**Fig. 6:**
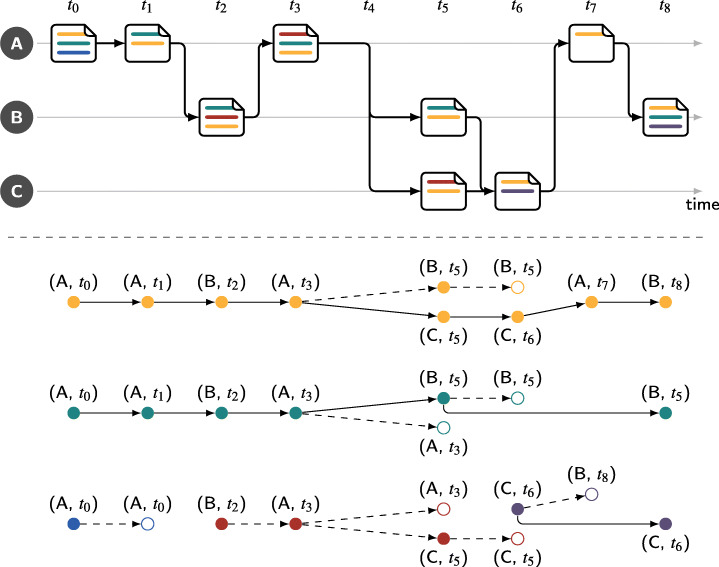
The extraction of line-editing DAGs from a code file edited by three developers (A, B, and C). Individual lines and their position in the file are colour coded. Forks and merges in the development process are indicated through the arrows between the versions of the file. Line editing paths are unique paths from a root to a leaf node in the individual DAGs

Figure 6 illustrates the extraction of line-editing DAGs—the first step in the extraction of line-editing paths. Here, a single file is edited by developers A, B, and C. Lines are colour-coded and are modified over time and consecutive versions of the file are linked by edges.

When editing a file, individual lines can change their position, e.g. when new lines are added/removed in the beginning of the file, or the source code is reorganised. Examples for this can be seen for the yellow line between *t*_0_ and *t*_1_ as well as the red line between *t*_2_ and *t*_3_. By applying git blame with the -C option to every version of the file, git2net is able to detect and track the positions of lines through such changes. Lines can further be deleted as shown by the example of the blue line between *t*_0_ and *t*_1_. When this occurs, an additional node indicating the removal is added to the respective DAG.

Particularly challenging to track are cases when developers fork a repository to work on multiple different versions (branches), later merging the combined changes back to a single version. An example for this is shown between *t*_3_ and *t*_6_. Here, both developer B and C create a personal copy of the version last modified by A, proceeding to make individual changes as shown in *t*_5_. In the line-editing DAGs, this can result in multiple concurrent versions of a line as shown by the example of the yellow line where at *t*_5_ both a version by B and C exists. In other cases lines can be removed from one branch while still remaining in the alternative version (cf. the green and red line). When merging two versions of a file, the author of the merge decides which changes to adopt from each branch. In addition to this selection, we found a number of cases in which developers contributed additional code to a project while performing a merge. An example for this is the purple line introduced in the merge commit by C at *t*_6_. To allow the extraction of line-editing paths, we added the functionality to mine changes made in merges to git2net. Note that, with development taking place on both branches before a merge, all files modified on either branch since the original fork need to be analysed. Given that in most cases no active changes are made during a merge, the relative computational effort compared to other commits is very high when analysing co-editing relationships. However, in the case of line editing paths their consideration is crucial as, by design, merges contain a large number of ending paths in the line-editing DAGs. Examples for this are the green and red lines that are not adopted when making the merge at *t*_6_.

Finally, using the -C option of git blame, git2net allows to detect if a line added to a file was copied from any other version of any file in the repository. In the figure, two examples for this are shown with the green and red line in *t*_8_ neither of which existed in the version of the file at *t*_7_. Tracking copies of lines is very powerful as it allows the analysis of reorganisation efforts, e.g. when a functions in a file are distributed to potentially multiple other locations.

After the extraction of line-editing DAGs, line-editing paths can be obtained as the set of unique paths from any root node to any leaf node in the set of line-editing DAGs. We expect line-editing paths to provide highly valuable insights into information exchange and knowledge transfer, e.g. the adoption of new coding techniques, in both open-source as well as proprietary software development teams.

### Usage of git2net

git2net comes as a python package that can be installed via the python package manager pip. During the installation, all dependencies, which consist of the python packages pandas, python_Levenshtein, pyDriller, tqdm, and pathpy, will be installed automatically. git2net runs on all major operating systems and has been tested under Windows, Mac OS X, and Linux. Assuming that the git repository that shall be examined has been cloned to a directory repository, our tool can be launched by the command
 where database indicates the sqlite database file where the results will be stored. An optional parameter –exclude can be used to pass a text file that contains paths of files or directories in the repository tree that shall be excluded from the analysis. In our own analyses of a large proprietary software project, this function has proven crucial to exclude directories containing large binary files or external Open Source software dependencies that would considerably distort the analysis. While the analysis of co-edited code uses the line-based approach described above by default, an optional command line switch –use-blocks can be used to use the block-based extraction of co-editing relations instead.

In addition to the command line interface outlined above, git2net provides an API that can be used for the development of custom repository mining scripts. In particular, the API provides methods that allow to extract co-edit relations from individual commits that can be passed as PyDriller objects. It can further be used to augment the analysis of edited code blocks by advanced text mining and code analysis techniques. We provide detailed inline documentation as well as a tutorial detailing how to get started using git2net.

In order to generate network projections based on a database of co-edits, git2net can be launched with the command
 where type can be coedit, coathor, bipartite, line_editing, or commit_editing. Depending on the choice, git2net generates a projection of the co-editing database in terms of a temporal co-editing network (cf. Fig. 4), a bipartite network linking authors to files, or a directed acyclic co-editing graph (cf. Fig. 5) respectively.

All networks can be exported in a csv-based format that can be read by popular network analysis packages like pathpy (Scholtes 2017a), igraph (Csardi and Nepusz 2006), NetworkX (Hagberg et al. 2008), graph-tool (Peixoto 2014), and Gephi (Bastian et al. 2009). Time-stamped co-editing networks can further be exported in a format that can be read by the dynamic network analysis and visualisation packages ORA (Carley and Pfeffer 2012) and pathpy (Scholtes 2017a) via the provided API. Moreover, all networks can be exported in terms of dynamic and interactive d3js visualisations, which directly run in any HTML5-compliant browser.

### Experimental Evaluation of Scalability

We conclude this section by an experimental evaluation of the scalability of git2net. In particular, our tool facilitates the analysis of large repositories thanks to the automatic utilisation of multiple processing cores. By default, git2net uses all available processing core, creating multiple child processes that extract co-edits from independent commits in parallel. An optional command line parameter –numprocesses N further allows to limit multi-core processing to at most *N* processing cores. By setting *N* to one, multi-core processing can be deactivated entirely. Similarly, the API exposed by git2net provides parameters that can be used to control multi-core processing.

In order to evaluate the scalability gains provided by the parallel processing model, we performed an experiment using real-world data. We specifically cloned the git repository of the Open Source software igraph (Csardi and Nepusz 2006) and used git2net to extract line-based co-editing relationships. We then measured the time needed to analyse the full git history with close to 6,000 commits and approximately 35,000 file edits over a period of 14 years^2^. We repeated this experiment multiple times, using different numbers of processing cores on a recent 16 core desktop processor^3^.

**Fig. 7:**
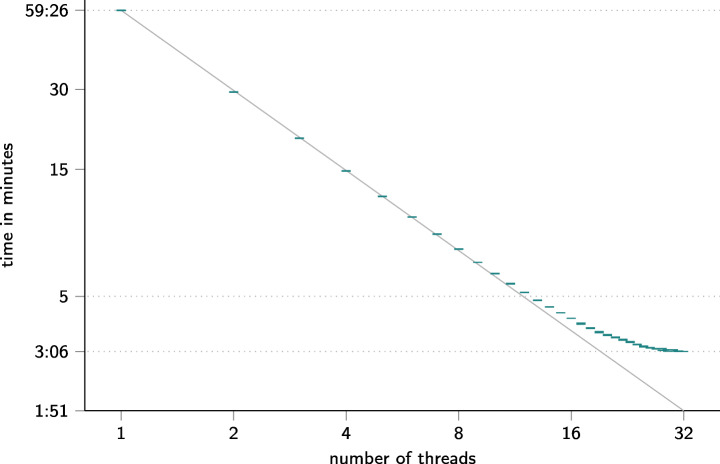
Time required to analyse the git repository of the software package igraph (Csardi and Nepusz 2006) for different numbers of parallel processing threads. Both axes are logarithmic. Bars show the mean and standard deviation of three runs. The grey line shows a perfect linear scaling based on the time required by a single-threaded analysis. Note that the time required to mine a repository with git2net is subject to change with future feature additions as well as performance improvements. The shown results are therefore to be interpreted as indicative for scaling not for absolute times

Figure 7 shows the time required to extract all co-editing relationships from the repository of igraph (y-axis) plotted against the number of processing threads (x-axis). Up to the number of physical processing cores of the machine (16) we observe an almost perfect linear scaling of processing time, cutting down processing time from close to one hour (single-threaded) to less than 5 minutes. Starting from 16 processing cores we observe deviations from the linear scaling that are likely due to the synchronised writing to the sqlite database. This deviation from the linear scaling is naturally intensified as we exceed the number of physical processing cores, additionally utilising logical cores exposed through Intel’s implementation of HW-based multi-threading.

## Exemplary Co-Editing Analysis of an Open Source and Proprietary Project

Having discussed the implementation, usage, and scalability of our tool, we now demonstrate its usefulness through four short exemplary studies of real-world software projects. We apply git2net to the master branches (i) the GitHub repository of the Open Source network analysis software igraph (Csardi and Nepusz 2006), and (ii) a large git repository of a proprietary software project obtained via an industry collaboration with the software company Genua. We specifically demonstrate (A) the construction of different static network projections capturing co-editing, co-authorship, and code-ownership relations, (B) a comparative study of fine-grained co-editing networks vs. coarse-grained co-authorship networks generated at the level of files, (C) the analysis of dynamic co-editing networks by means of temporal network analysis techniques, and (D) a comparison of temporal co-editing patterns between an Open Source and a proprietary software project. These case studies should be seen as seeds for future work that demonstrate the usefulness of our approach rather than as conclusive analyses. To support such future studies, the co-editing relationships extracted from the Open Source project igraph are available on zenodo.org (Gote et al. 2019a).

### Static Network Projections

**Fig. 8:**
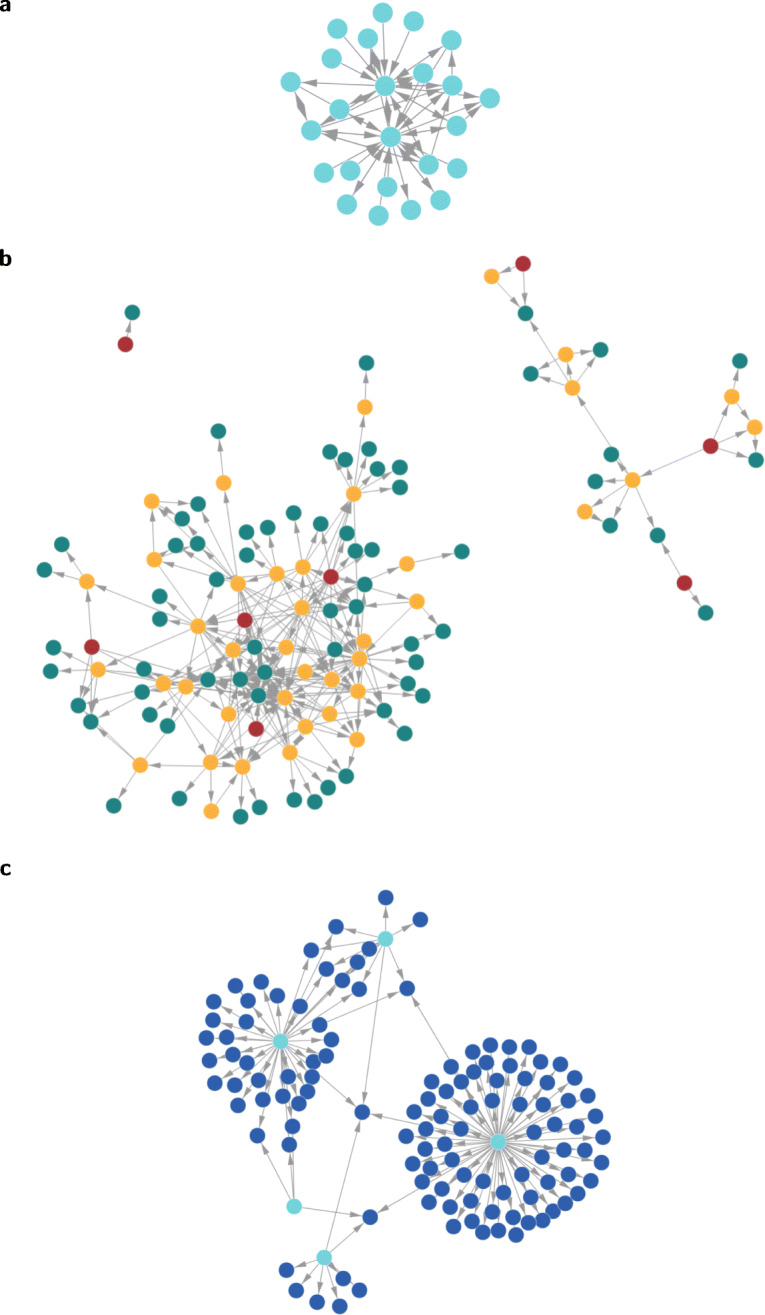
Three examples for time-aggregated collaboration networks generated by git2net based on co-editing relations in igraph project: a shows a time-aggregated, static, directed network of co-editing relations. Each node represents one developer, while a directed link (*A*, *B*) indicates that at some point in the development history developer *A* edited at least one line of code previously written by developer *B*. b shows a directed acyclic graph of edits of the source code file flow.c. Nodes represent commits by developers. Root nodes with in-degree zero are marked in red, leaf nodes with out-degree zero are marked in green, intermediary nodes are marked in red. c shows a bipartite network linking developers (lightblue) to the files that they edited (blue)

To demonstrate our tool, we illustrate the three different network projections introduced in Section 3, using the co-edit information extracted from the public git repository of the network analysis package igraph (Csardi and Nepusz 2006). The resulting networks are shown in Fig. 8.

Figure 8a shows a static co-editing network where nodes represent developers. For this initial demonstration we employ a time-aggregated projection, i.e. we use time-stamped co-editing relations (*v*, *w*;*t*) capturing that at time *t* a developer *v* edited code originally written by developer *w* to construct a time-aggregated graph *G*(*V*, *E*) where (*v*, *w*) ∈ *E* iff ∃*τ* : (*v*, *w*;*τ*). Git records both the committer and commit time, as well as the author and author time for all commits. While git2net extracts both, we use the author information as basis for the analysis. This choice is made as many popular projects start their development outside of GitHub and are only pushed there at a later point in time. This push is usually done by a single developer who then appears as the committer of all commits prior to the push. However, the author information is usually retained from the original commit outside of GitHub and thus points to the developer responsible for writing the code in the commit.

Co-editing networks allow us to study how code is developed in software projects. Particularly, they show how developers directly collaborate with others on individual lines of code. Note that we do not make any claims about direct communication (outside the code) between the two developers as these can vary between both for different lines and projects. However, if a project allows the assumption that collaboration on a line also requires additional external coordination efforts, communication requirements between pairs of developers can be derived. The directionality of links in the resulting time-aggregated projection further allows us to distinguish between team members with different roles: Nodes with zero in-degree, i.e. developers with no incoming co-edit relations, have never contributed code that was subsequently edited by other developers. Nodes with zero out-degree, i.e. developers with no outgoing co-edit relations, have never edited code that was originally authored by other developers. Such a maximally simple static projection can thus give a first “birds-eye” view of the collaboration and coordination structures in a software developing team. It highlights pairs of developers who exhibit strong mutual co-editing relations as well as pairs of developers working independently. This analysis can be refined by taking into account the time stamps of co-editing events, which we will do in Section 4.3. In Section 4.2 we further discuss the difference between file-based co-authorship networks considered in prior works and the static projection of a fine-grained line-based definition.

Apart from co-editing relations between developers, in Section 3 we have argued that git2net also provides a new perspective on the history of commits modifying a *given* file in the repository. In particular, this information can be used to a construct a directed acyclic graph of commits, where a link (*v*, *w*) in the graph indicates that commit *w* edited a region of source code originally contributed in commit *v*. Hence, each path from a root node *r* to a leaf node *l* in the resulting directed acyclic graph can be interpreted as a time-ordered sequence of commits that transforms code originally introduced in commit *r* into the “final” version contained in *l*. We highlight that this projection is different from commonly studied commit graphs, which link each commit to their parent commit independent of whether there is an overlap in the edited code. Figure 8b illustrates this idea. It shows the directed acyclic graph of commits for the source code file flow.c in igraph (Csardi and Nepusz 2006). Root nodes (with in-degree zero) in which the original version of a region of source code was committed are shown in red, while the commits containing the “final” version of code regions (out-degree zero) are highlighted in green. Intermediary nodes (yellow) represent commits that have both (a) edited code originally contributed in a previous commit and (b) contributed new code that is being edited in a subsequent commit. The analysis of such directed acyclic graphs can give insights into the complexity of code edits and their distribution across the team or across time. They further provide a novel abstraction that can be useful for the comparison of software artefacts, development processes, or projects.

In order to make it easy to reproduce file-based definitions of co-authorships used in the literature, git2net finally supports the construction of networks linking developers with the files that they have edited. The time-aggregated bipartite network resulting from the file edits made in the year 2016 for the project igraph is shown in Fig. 8c. Apart from being a basis for the construction of file-based co-authorship networks, this simple representation can give a coarse-grained view of code ownership and the distribution of contributions across the development team.

### Comparison of Co-editing and Co-authorship Networks

As outlined in Section 2, the analysis of co-authorship networks that capture which developers have contributed to the same files has received significant attention. At the same time, recent works have argued for more fine-grained definitions of collaboration networks, using e.g. function points or code lines (Joblin et al. 2015; Scholtes et al. 2016). We contribute to this discussion and investigate the differences between a line- and a file-based approach to construct developer collaboration networks. Our results show that (i) this choice of granularity has considerable influence on the resulting network topologies, (ii) that the resulting differences are project-dependent, and (iii) that the differences between the resulting networks exhibit temporal inhomogeneities.

For our analysis, we first use git2net to extract (a) a file-based co-editing network *G*_*f*_ (which for simplicity we call co-authorship network), and (b) a line-based co-editing network *G*_*l*_ for the Open Source project igraph as well as for a large proprietary software project. For both networks, we compare the time-aggregated projections (constructed as described in Section 4.1) and the sequence of networks obtained via a rolling window analysis. For each time window (as well as for the time-aggregated network), we then quantitatively assess the difference between *G*_*l*_ and *G*_*f*_. We first observe that the set of nodes in both networks is necessarily the same. As a maximally simple approach to assess the difference between the two networks, we can thus calculate $\delta := \frac {m_{f}}{m_{l}}$, where *m*_*f*_ and *m*_*l*_ are the number of links in the file-based co-authorship network and the line-based co-editing networks, respectively.

**Fig. 9:**
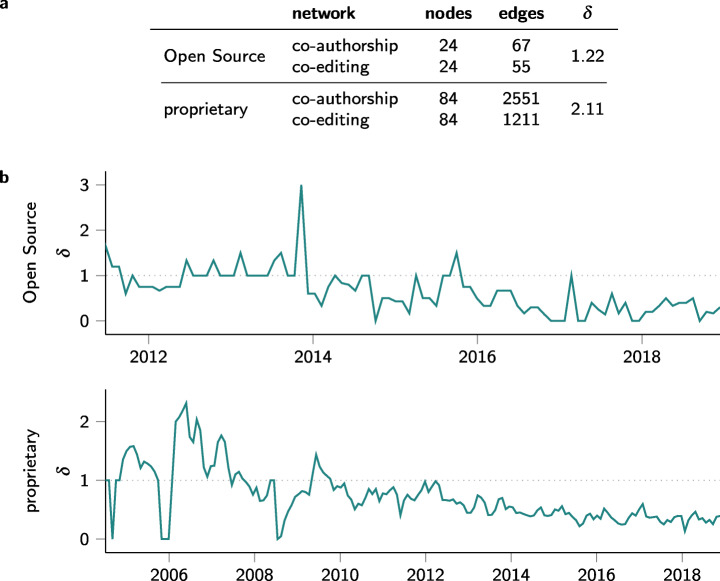
Comparative analysis of file-based co-authorship vs. line-based co-editing networks. a Number of nodes and edges of networks aggregated over the entire project duration. Here, the co-authorship network counts more relationships as it considers all possible indirect collaboration, whereas co-editing networks consider only collaboration directly recorded in git. b Proportion of edges in both networks over a moving 90 day window. Here, the co-authorship network frequently does not display links present in the co-editing network, as with co-editing links interactions with developers not contributing code in the present time window can be considered

Figure 9 shows the result of this analysis. Figure 9a confirms that the file-based co-authorship network does not resolve where in the file edits take place, leading to a significantly higher number of links compared to the co-editing network in both projects. These differences are present, as co-editing networks only account for direct relationships of developers editing the same line, whereas co-authorship networks also consider all possible indirect collaboration of developers editing the same file. Depending on the research question as well as programming language analysed both approaches have their own merit as the true interaction requirements likely lie somewhere in between. We highlight, however, that next to direct co-editing relations *git2net* also extracts the line number in which the change takes place. This fine-grained analysis therefore allows to define links between developers where the weight of the link is a function of the distance between the location of the edited lines.

Figure 9b highlights the temporal dimension of these differences. It shows the time-evolving difference between the two network abstractions, using a 90 day moving window. For each window, the difference *δ* between the two networks is reported. Importantly, we observe time windows where *δ* < 1, which indicates that the line-based co-editing networks feature additional links over the file-based co-authorship network. This is due to the fact that a file-based (temporal) co-authorship network does not consider commits to files made outside the time window currently analysed. However, our detailed analysis of co-edit relations can nevertheless identify that at time *t* within the time window developer *A* has edited code originally authored by developer *B* in a commit outside the time window. We argue that neglecting this relation introduces the risk of omitting the need of collaboration or coordination associated with a commit occurring at time *t*. This subtle but important difference highlights the limitations of a simple file-based extraction of collaboration networks and showcases the differences and advantages of our approach.

### Analysis of Temporal Co-Editing Networks

**Fig. 10:**
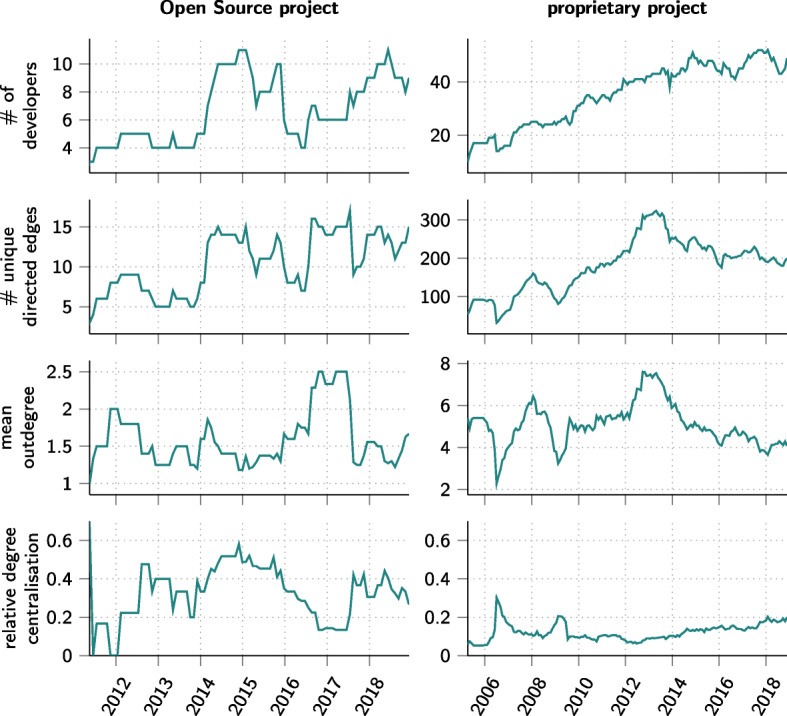
Time series of different (network-analytic) measures for the time-stamped co-editing networks of an Open Source (left) and proprietary software project (right). Results were generated using a rolling window analysis with a window size of 365 days and 30 day increments

A major advantage of git2net is its support for the extraction of *dynamic* co-editing networks with high temporal resolution. To showcase the benefits of such a temporal analysis for the two projects mentioned above, we have used git2net’s python API to extract a time-stamped co-editing network from the repositories of the two projects mentioned above. We then used the temporal network analysis package pathpy (Scholtes 2017a) to apply a rolling window analysis, which provided us with a time series of network analytic measures. Figure 10 shows the resulting time series for four measures both for the Open Source project igraph as well as the proprietary software project. The first row gives the number of developers working on the projects in a 365-day sliding window. The number of unique co-editing relations between these developers, shown in the second row, can be used to proxy the amount of collaboration on joint code regions taking place in a project in a given time window. We observe that the number of such collaborations relative to the number of developers is considerably higher for the proprietary software project compared to the Open Source project. This finding is further corroborated by the mean out-degree of nodes shown in the third row. This suggests that on average developers in igraph edit the code of one to two other developers, while for the proprietary software project each developer has to coordinate his or her changes with four to eight other team members. It is a remarkable finding for the proprietary software project that both the number of unique directed edges and the mean out-degree decline from 2013 onward, despite the growing number of developers. This could mark a change in the software development processes and/or the social organisation of teams. While a first feedback from the project managers suggests that this could be related to a change in the adoption of an agile development model, testing this hypothesis requires a separate in-depth study. Finally, in the fourth row in Fig. 10 we report the evolution of normalised (total) degree centralisation over time (Freeman 1978). A minimum value of zero indicates that all nodes in the network have the same degree, while a maximum value of one corresponds to a perfect star network where all nodes except a hub node have degree one. We find that igraph exhibits considerably larger degree centralisation than the proprietary software project, which is likely related to previous findings of highly skewed distributions of code contributions in Open Source projects (Scholtes et al. 2016; Mockus et al. 2002; Lin and Whitehead 2015).

### Editing of Own vs. Foreign Code

In a final experiment, we showcase how git2net can be used to analyse temporal co-editing patterns in software development teams. To this end, we extend our analysis of the mere *topological* dimension of co-editing relations performed in previous sections, to use additional information on the Levenshtein distance associated with these relations. The Levenshtein distance between two source code versions captures the number of characters one has to type to transform one string into another string. It has been used as a proxy for development effort associated with commits (Scholtes et al. 2016). Extending this approach, an interesting aspect of our methodology is that it allows us to distinguish between (i) the cumulative Levenshtein distance of code edits made in a developer’s *own* code and (ii) the cumulative Levenshtein distance of edits made in *foreign* code, i.e. code originally written by other developers. This enables us to calculate, for each time window in the commit history of a project, the relative proportion of development effort falling into these two categories.

**Fig. 11:**
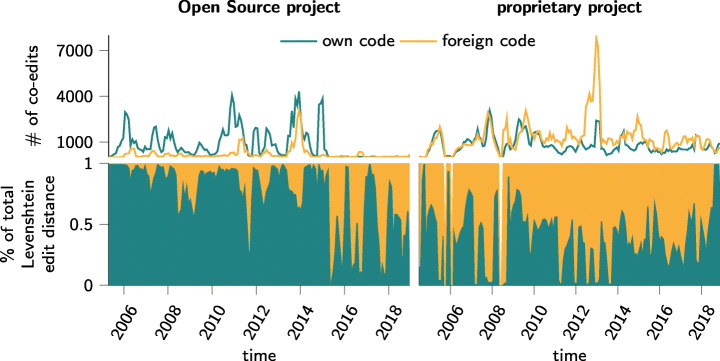
Editing of own and foreign code for Open Source and commercial project over time. The total number of edited blocks is shown above whereas the bottom figures show proportions of the total Levenshtein edit distance. Results are computed on a 90 day rolling window with 30 day increments

Figure 11 shows the result of this analysis for the two projects introduced above, where the top-part of the figure reports the total number of (unweighted) co-edit relations, while the bottom part shows the relative proportion of the total Levenshtein distance of own code changes vs. foreign code changes. This analysis highlights considerable project- and time-dependent differences. For the Open Source project igraph, during a first phase from 2006 to 2015, the majority of code edits take place in code previously written by the same developer. This indicates a strict notion of code “ownership”, where developers rarely touch code written by others. For the proprietary software project we observe a completely different dynamics, where for the majority of time windows development effort is dominated by *foreign* code edits. We hypothesise that this finding is likely related to code changes triggered by the specific implementation of the code review process in the proprietary software project (Beller et al. 2014). This finding highlights a specific research question that can be addressed with our tool in future work.

## A Large-Scale Analysis of Coordination Overhead in Software Teams

Leveraging fine-grained co-editing networks and code ownership information generated by git2net, we conclude our article with an empirical study of factors that influence the productivity of developers in collaborative software projects.

Specifically we aim to look at a mechanism behind the Ringelmann effect from social psychology stating that members of larger groups tend to be less productive (Ringelmann 1913). This effect is strongly linked to Brooks’ Law of Software Project Management postulating that adding developers to a late project makes the project later (Brooks FP 1975). As discussed in Section 2.2, literature points to growing coordination overhead as one factor that drives this decrease in individual productivity in software teams. However, this interpretation of the results is also relying on the (untested) hypothesis that the editing of code from other developers imposes an actual overhead that is associated with a (measurable) decrease in productivity. With the following study, we take a next step in understanding the causal link between (i) co-editing patterns and code-ownership and (ii) developer productivity in software teams. Specifically, we take a microscopic perspective that leverages detailed information on co-edited lines and commits provided by git2net. In particular, we base our study on the following hypothesis about the link between code ownership on developer productivity:

### Hypothesis

Developer productivity is higher when developers edit code previously written by themselves compared to code written by other developers.

In the remainder of this section we will use statistical methods to test this research hypothesis in six OSS projects developed on GitHub. The selected projects cover a wide range of sizes (cf. Table S1)) and topics ranging from the network visualisation and analysis library igraph to the Linux kernel. An overview of the six projects is given in Table 1. In total, our data set covers more than 1.2 million commits by more than 25,000 developers. Referring to the analysis laid out in Section 4.4, we first use git2net to extract the temporal co-editing patterns from the time-ordered sequence of all commits. Moreover, for each edited line within the files contained in a commit, git2net enables us to infer whether the line in question was previously edited by a different developer. By calculating the Levenshtein edit distance of each edited line we can associate the relative fraction of the Levenshtein edit distance in own vs. foreign code for each individual commit. Finally, the sequence of time-stamped commits of individual developers allows us to give an upper bound for the development time of a commit. We are particularly interested in the question to what extent the editing of code previously written by one or more *other* developers takes, on average, longer than the editing of own code—i.e. code written by the author of the edit her- or himself.

**Table 1:** Short description of the Open Source Software projects used in the case study

Repository	Description
igraph/igraph	A network visualisation and analysis library
bitcoin/bitcoin	The source code enabling the digital currency Bitcoin
libav/libav	Cross-platform audio and video processing tools
FFmpeg/FFmpeg	Cross-platform solution to record, convert and stream audio and video
gentoo/gentoo	The Gentoo Linux distribution
torvalds/linux	The kernel of the GNU/Linux family of operating systems

All projects are developed on GitHub and can be found under the given repositories

For the purposes of this study, we quantify productivity as the amount of code produced in a given time interval, where code production is measured as the number of characters produced or changed. We specifically do not assume that the amount of code produced is an indicator for the quality of the software. In line with literature in management and organisational studies we explicitly distinguish between *productivity*, which is a measure of the amount of artefacts produced in given period of time, and *performance*, which additionally includes a quality dimension of the produced artefacts (Tangen 2005). In other words, a highly productive developer can perform poorly and vice-versa. Exclusively focusing on this notion of productivity (and excluding the more elusive concept of performance) our study aims at a mechanistic explanation of recent studies that have evaluated changes in the amount of code produced per developer with the change of the team size.

We quantify the time in which all the production related to a commit takes place based on the inter-commit time between two consecutive commits of the same developer (Vasilescu et al. 2013; Di Bella et al. 2013). Hence, the inter-commit time is the time difference between any two consecutive commits in the ordered sequences of commits made by the same author. Note that we do not impose an upper limit for the inter-commit time between two subsequent commits of the same author above which such commits would no longer be considered consecutive. We argue that, despite being limited, this is the best assessment of development time that we can derive directly from the repository at large scale. Clearly, we have no knowledge whether developers performed other tasks during that time, but we rest our analysis on the assumption that this affects all commits equally, independently of whether they take place in own code or not. Also we highlight that we do not use the inter-commit times to evaluate the productivity of developers, but rather comparing own code vs. foreign code commits. As such our analysis assumes that there is no difference in terms of parallel tasks by developers between own and foreign code edits.

### Relation Between Foreign Code Editing and Team Size

**Fig. 12:**
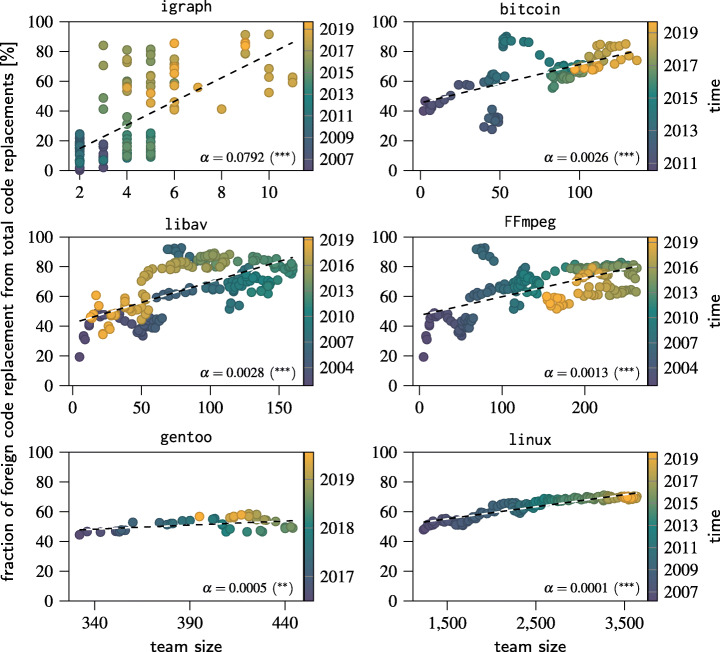
Fraction of foreign code replacements from total code replacements for different team sizes. Colours show the development over time. The parameter *α* represents the slope of a linear *y* = *αx* + *β* for the the plots. The asterisks indicate *p* < 0.05 (*), *p* < 0.01 (**), and *p* < 0.001 (***) for the slope being positive

To connect our hypothesis to the Ringelmann effect, we first confirm that coordination requirements indeed increase with the size of the development team. To measure coordination requirements within a team, we consider the edits made to lines of code. Here, we proxy the amount of coordination relative to the team size by the total Levenshtein distance of edits made to foreign code compared to own code. In other words, we assume that it requires less coordination to edit own code than to edit code last edited by other developers. Figure 12 shows the coordination requirement relative to the team size for a rolling window analysis with a window size of 295 days and a time increment of four weeks. The window size was selected based on the finding of Scholtes et al. (2016) that after 295 days of inactivity, the probability of a subsequent commit of an Open Source Software developer is less than 10% and hence the developer should no longer be considered as member of the development team. The dashed line shows a linear model of the form *y* = *αx* +β fitted to the data. Colours show the development of both team size and coordination requirements over time.

The significant and positive slopes for all fits confirm that coordination requirements increase with team size for all considered projects. We further find that the effect size is reduced for larger teams. This is likely due to the creation of modular structures in larger projects, however it is outside of the scope of this work to confirm this. Finally, we note that there appears to be no lock-in effect at higher levels of coordination as the proportion of foreign code replacements declines again with a reduction in team size as can be seen for libav, FFmpeg and bitcoin.

### Feature Selection

#### Productivity

In our research hypothesis we state that productivity (which in the following we refer to as *prod*) is higher if the developer making an edit is also the author of the original (edited) code. To quantitatively test this hypothesis we define productivity as an input-output relationship (Tangen 2005), defining output as the amount of code written by a developer. As we are only interested in situations where existing code was edited or extended, we measure the output as the Levenshtein edit distance for each edit (*lev*). With a set of multiple edits forming a single commit, the inter-commit time between two consecutive commits (*ict*) serves as upper bound for the time required to produce the edits. With this, we define developer productivity as $prod = \frac {lev}{ict}$.

We emphasize that our definition of productivity is based on the cumulative changes made to the source code over time, rather than only counting contributions to the final version of the source code. We note that both approaches give interesting insights into productivity and the contribution of developers to a software project. However, for the purpose of our study we chose to consider all changes towards the final version as we are interested in the collective contributions of all developers over time.

#### Own code

To determine if the edit was made in own or foreign code, we compare the current and previous author of the code as identified by git2net. In addition to code ownership, there are multiple other factors that could influence the productivity of a developer. Particularly, we want to control for the complexity and the type of the change, as well as the developer’s experience and the type of the project.

#### Complexity

We expect the complexity of an edit to have a—supposedly negative—impact on productivity. Moreover, we can imagine situations where code with higher complexity is more likely to be edited by multiple developers, resulting in a confounding factor that could explain an observed negative correlation between productivity and the editing of foreign code that we must control for. To this end, we proxy the complexity of the change by looking at the cyclomatic complexity (*cyc*) McCabe (1976) of the file the edit was made in. We additionally measure the length of the file in terms of the number of lines (*nol*) as well as the size of the project in terms of the number of commits (*noc*_*t*_), correcting for all three measures in our analysis.

#### Type of change

During the development of git2net, we observed that those changes that are made in multiple files within a single commit are often similar. As these are often relatively simple operations we can expect those edits to be generally faster, which highlights another possible confounding factor that we must control for. For this, we include the number of files edited in a commit (*nof* ) in our analysis. In addition, a commit can be made to fix bugs in a recently introduced feature or to change the functionality of code already present in the project for a longer period of time. We aim to capture this difference by considering the time since the previous edit of a line before the current edit (*tpe*).

#### Developer experience

An important factor that is likely to influence the productivity of a developer is her or his experience. Since we have no information about developers outside the current project, we measure developer experience with respect to the project in question. We proxy the experience through the number of commits the developer made to the project (*noc*_*d*_) at the time of the current commit. In addition, the time since the developer’s first commit to the project (*tfc*) is considered as an additional control variable.

#### Type of project

Finally, there are Open Source Software projects like Linux on which many developers work as full-time job, whereas others are side projects that developers mainly work on during their free time, e.g. on weekends. We account for these differences through a binary variable indicating if an edit was made during a weekday (*wkd*).

Many of the features introduced above can be measured at the level of individual lines of code. However, inter-commit times are only available at the commit level. Therefore, all edits relating to a single commit need to be aggregated, which we can achieve either through a simple sum or a weighted summation that accounts for the Levenshtein edit distances of individual edits. Wherever multiple weights are possible all options were explored. Overall, this leads to the set of features shown in Table 2.

**Table 2:** Description of all features obtained for the analysis

Feature	Description
*lev*	Total Levenshtein distance (in characters) though code replacement in commit
*ict*	Time (in hours) since last commit made by the current developer
*prod*	Developer productivity computed as $prod = \frac {lev}{ict}$
*own*	Fraction of *lev* made on own code
*cyc*_*l*_	Cyclomatic complexity of edits weighted by Levenshtein edit distance
*cyc*_*f*_	Cyclomatic complexity of edits weighted by number of unique files in commit
*nol*_*l*_	Number of lines in edited files weighted by Levenshtein edit distance
*nol*_*f*_	Number of lines in edited files weighted by number of unique files in commit
*noc*_*t*_	Total number (in thousands) of commits in the project at the time of the commit
*nof*	Number of unique files edited with the commit
*tpe*_*l*_	Time (in years) since previous edit of line weighted by Levenshtein edit distance
*tpe*_*e*_	Time (in years) since previous edit of line weighted by number of edits in commit
*noc*_*d*_	Number commits (in thousands) made by the developer at time of the commit
*nfc*	Time (in years) since the first commit of the developer making the commit
*wkd*	Logical variable indicating if the commit was made on a weekday

### Data Collection

The data was collected by cloning the six repositories and subsequently applying git2net to mine the relevant co-editing relationships. git2net employs a parallel processing model, nevertheless, a git blame operation needs to be executed and the result must be analysed for each file modified in a commit. This makes processing large commits particularly expensive, which is why we needed to filter out a small number of commits that were modifying more than 1000 files each. These large commits primarily fall into two classes: The majority are merge commits, which unlike commits with a single predecessor cannot be processed through a single *diff*. Therefore, all files in the *diffs* of both parents need to be analysed for all line edits in the merge commit. As merges are often made between different branches of a repository, this can lead to a very large number of files included in the *diffs*. We argue that excluding large merges from our analysis will not affect our results as, generally, merge commits serve to combine different versions of commits made in previous commits and do not contain any new contributions. Through direct inspection of the other filtered commits we found that these are mostly the result of search and replace operations across a large number of files—e.g. replacing http through https^4^. As these are only a small fraction of commits and such changes often do not take any significant understanding of the code in which they take place, we have opted to not consider them.

As shown in Table S1, the commits removed based on the filtering procedure mentioned above represent less than 5% of the total commits of the six projects individually. In the following, the data required to test the hypothesis was selected from the databases generated by git2net. We further perform an additional data cleaning step, which we describe in detail in the Supplementary Material (see Section S1). In particular, we discuss which edits were used in our analysis, how name disambiguation was considered for commit authors, as well as additional steps necessary due to project-dependent differences in the usage of git. We describe that especially linux developers frequently make multiple commits with very small inter-commit times—often less than one second—at the end of a development process. In order to avoid that such near-zero inter-commit times distort our analysis, we aggregate the corresponding commits into a single *contribution*, which is the subject of the following analysis (details in Section S1).

### Results

As a precursor for the more sophisticated analyses that we will perform in later steps, we first check whether commits in which the majority of edited lines are in own code are developed faster (i.e. the productivity for those edits is higher) than those for which the majority of edited code was previously written by other developers. In this first step of our analysis, we merely test whether the data contains a pattern that justifies advanced tests in which we will control for potential confounding factors. We use a Wilcoxon signed-rank test to compare the productivity distributions of those contributions with *own* ≥ 0.5 to the productivity distribution of contributions with *own* < 0.5 The results in Table 3 shows that, for all of the six considered projects, we can safely reject the null hypothesis that both samples are drawn from the same distributions against the one-sided alternative hypothesis that the productivity is higher for contributions in which the majority of edits occur in own code.

**Table 3:** Results of Wilcoxon signed-rank test. Alternative hypothesis: productivity on own code is higher

	igraph	bitcoin	libav	FFmpeg	gentoo	linux
*p*-value (greater)	**0.0000**	**0.0000**	**0.0000**	**0.0000**	**0.0000**	**0.0000**
median speed own	12.92	7.57	7.21	7.54	6.97	1.54
median speed foreign	2.37	3.38	3.40	4.61	5.51	0.79
mean speed own	91.10	77.82	79.23	68.56	47.42	38.94
mean speed foreign	53.09	62.44	67.29	61.58	41.36	29.56

Values in bold highlight the result of the Wilcoxon signed-rank test

**Fig. 13:**
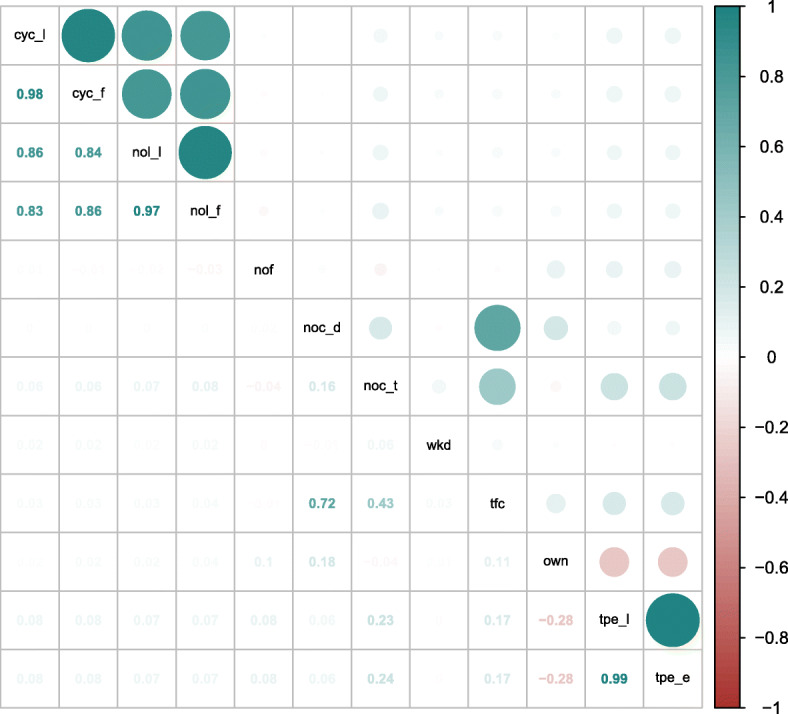
Spearman’s rank-order correlations of pairs of features (indicated in the diagonal entries) for the linux project *before* feature selection

In the following, we provide a more thorough analysis of the multiple factors that influence the productivity of developers based on a multiple linear regression model. We first define the regression model that we will use. In Section 5.2, we have explained all features as well as the motivation why we include them as control variables. Ideally, we aim to include all potential confounding effects as controls parameters in our statistical analysis, hence isolating the effects of code-ownership and co-editing patterns that are of interest in the context of this study. However, some of the features introduced above only differ by their aggregation weights and thus bear a large potential for collinearity, which can invalidate the results of the regression analysis. As a first step, we thus perform a feature selection process, which we illustrate in the following using data from the linux kernel development project. The corresponding results for all other projects included in our analysis are presented in the Supplementary Material (Section S3). Figure 13 shows the Spearman’s rank-order correlations for all pairs of available features for linux.

**Fig. 14:**
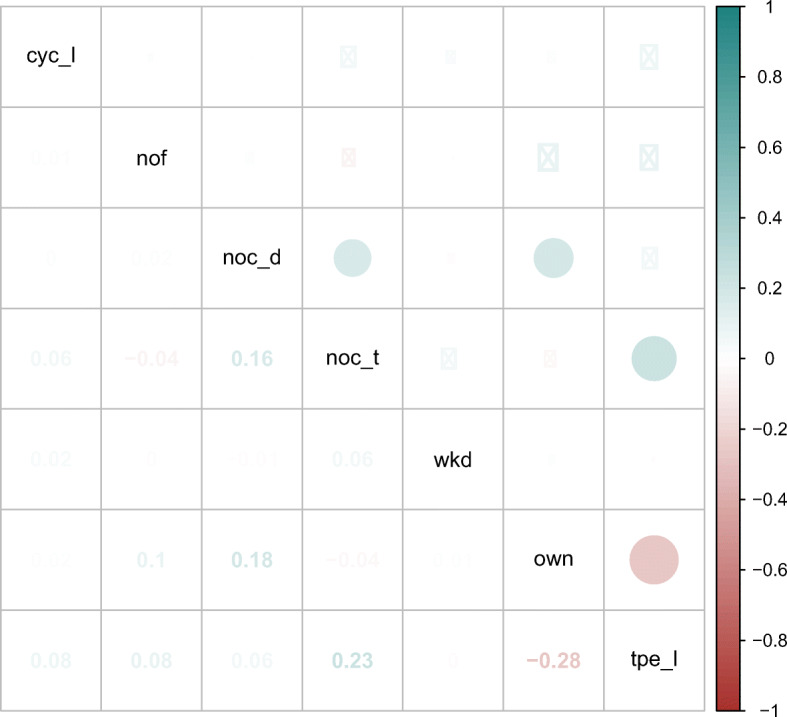
Spearman’s rank-order correlations of pairs of features (indicated in the diagonal entries) for the linux project *after* feature selection

We find that *cyc*_*l*_ and *cyc*_*f*_, *nol*_*l*_ and *nol*_*f*_, as well as *tpe*_*l*_ and *tpe*_*e*_, exhibit high levels of correlation. This is due to the fact that those features only differ by the method by which the line-level measures are aggregated at the level of commits. Moreover, the cyclomatic complexity (*cyc*_*l*_ and *cyc*_*f*_) of code is strongly correlated with the number of lines in the edited file (*nol*_*l*_ and *nol*_*f*_). This is expected, as cyclomatic complexity is defined as the number of linearly independent paths through the file’s source code (McCabe 1976). Finally, we find a strong correlation between a developer’s number of commits and the time since the developer’s first commit. Regarding the method used to aggregate line-level measures at the commit level, we decide to always use a method that weights the measures by the Levenshtein edit distance. For the remaining results, we thus exclude *cyc*_*f*_, *nol*_*f*_, and *tpe*_*e*_. We further decided to include cyclomatic complexity rather than the number of lines, since cyclomatic complexity is an established metric that carries additional information about the complexity of code. Lastly, we include the number of commits, *noc*_*d*_ rather that the mere time since the first commit, as we expect the latter measure to contain less information about the actual development experience in a project. Figure 14 shows the pair-wise Spearman’s rank-order correlations of the remaining features for the linux project^5^.

To test our original research hypothesis while accounting for potential confounding effects, we define three multiple regression models that include the set of features selected as described above. We then employ a statistical model selection based on Aikake’s Information Criterion (AIC) to determine the most suitable model (Akaike 1974). In Table 4 we provide detailed definitions of the three multiple linear regression models that we consider in the following.

**Table 4:** Regression models

Name	Formula
LM	$prod \sim own + cyc_{l} + nof + noc_{d} + noc_{t} + tpe_{l} + nfc + wkd$
ME-	$prod \sim ~~~~~~~~~~~~ cyc_{l} + nof + noc_{d} + noc_{t} + tpe_{l} + nfc + wkd + (1 + noc_{d}~\vert ~author)$
ME+	$prod \sim own + cyc_{l} + nof + noc_{d} + noc_{t} + tpe_{l} + nfc + wkd + (1 + noc_{d}~\vert ~author)$

Formulas are given in the notation of the R mixed effect model library lme4 (Bates et al. 2014). The variable *author* is a factor variable distinguishing authors

The first model is a standard multiple linear model (LM) capturing a linear relationship between developer productivity and the fraction of own code edited, while controlling for other features. In the other two models, we additionally correct for the fact that we have multiple contributions by multiple developers that are likely to have heterogeneous characteristics (e.g. talent, intelligence, education, etc.) that we may not directly observe in our data. We control for this effect by means of a mixed effects model *ME* − that models the differences between developers by including an individual base productivity. To additionally account for the *observed* heterogeneity of developers in our data, we further include the developer experience (in terms of number of commits *noc*_*d*_) as an additional developer-dependent feature in our mixed effects model (see Table 4). Finally, we consider a mixed effects model *ME* + that additionally includes *own* as an independent variable.

We fit each of the three linear models to the data from each of the six projects individually and calculate the AIC to select the model that provides the best balance between explained variance and model complexity. To illustrate our approach, we show the detailed results of the model selection for linux in Table 5. The results for other projects are included in the Supplementary Material (Section S4).

**Table 5:** AIC as well as Chi-squared test for the three candidate models for linux

linux	Df	AIC	Chisq	Chi Df	Pr(>Chisq)
LM	9	11745.96	—	—	—
ME-	11	117.75	11632.21	2.0	0.0
ME+	12	0.00	119.75	1.0	0.0

For all projects, ME+ is clearly selected as the best model that minimises the AIC. This shows that despite the larger complexity of the mixed effects model, controlling for developer-specific features is essential even for projects like igraph with a small number of developers. Moreover, we find that including the feature *own* (in ME+) considerably improves the model fit for all of the six projects, even when account for the additional model complexity.

Table 6 shows the estimated parameters in the selected mixed effects multiple linear model ME+ in all six projects included in our case study.

**Table 6:** Regression results of ME+ for all projects

	igraph	bitcoin	libav	FFmpeg	gentoo	linux
*own*	27.02^**^	10.99^*^	15.86^***^	5.76^***^	6.28^***^	5.01^***^
intercept	21.43	36.59^***^	46.73^***^	41.12^***^	31.88^***^	19.14^***^
*cyc*_*l*_	0.05^***^	0.01^*^	0.0	− 0.0	− 0.35^***^	0.0^**^
*noc*_*t*_	3.68	− 0.53	− 0.71^***^	− 0.24^***^	− 0.04^***^	− 0.02^***^
*nof*	2.17^***^	1.23^***^	1.44^***^	1.72^***^	1.32^***^	1.46^***^
*tpe*_*l*_	0.11	1.64	4.99^***^	2.19^***^	14.13^***^	1.11^***^
*noc*_*d*_	0.28	4.23^***^	2.15^**^	1.46^***^	0.28^***^	1.82^***^
*wkd*	4.21	1.63	− 8.58^***^	− 3.91^**^	− 4.99^***^	− 2.16^***^

Note that we do not correct for multiple hypothesis testing since *own* is the only independent variable for which interpret the *p*-value in terms of statistical significance, while all other features are merely included as control variables. The asterisks indicate *p* < 0.05 (*), *p* < 0.01 (**), and *p* < 0.001 (***)

The results provide strong evidence for our hypothesis that the need to edit code previously written by other developers negatively affects developer productivity, even when controlling for potential confounds like code complexity, the type of a change, developer experience and project characteristics. In particular, we reject the null hypothesis that there is no effect of the feature own code (i.e. *own* = 0), assuming all other factors are equal for all projects. In summary, these findings substantiate the theory that the need to coordinate the efforts of increasingly large number of team members is a driving factor behind the Ringelmann effect in software teams. Moreover, the fitted parameters of our model allow us to estimate the size of the effect. As an example, for the linux project commits exclusively consisting of edits in code previously written by the same developer (i.e. *own* = 1) has – on average – a productivity value that is larger by 5.01 characters per hours compared to commits exclusively consisting of code previously written by other developers (i.e. *own* = 1), assuming that all other factors are equal. Considering the intercept value of 19.14 characters per hour for the linux project, this yields a relative productivity increase of more than 25*%*. For the five other projects we obtain relative increases in productivity between 14*%* (FFmpeg) and 128*%* (igraph). We thus find large project-specific differences in terms of how code ownership affects developer productivity. This potentially provides an additional microscopic explanation for the finding in Scholtes et al. (2016) that the magnitude of the Ringelmann effect widely varies across different projects.

## Future Work

We highlight three research directions that can be addressed using git2net.

Publicly available repositories cover a variety of different collaborative tasks, like software development, manuscript editing, web content management, etc. Kalliamvakou et al. (2016). The excellent scalability of git2net facilitates the efficient mining of a large number of such repositories and thus opens up an important new source of high-resolution data on human collaboration patterns. This approach is further facilitated by other tools such as GHTorrent (Gousios and Spinellis 2012) and World of code (Ma et al. 2019) that allow for efficient scanning for projects suitable to address the research questions at hand. The resulting set of projects can then be mined with git2net allowing fine-grained access to large sets of information on the Open Source Software development process. This is likely to lead to deeper insights into project success, knowledge transfer, the impact of project modularity and hierarchy, as well as project dependencies.

The fact that the resulting dynamic collaboration networks (including co-editing, co-authorship, developer-file, and line-editing networks) can be cross-referenced with project-related information (project success, organisational structures and project culture, developer roles, etc.) is likely to be of value for researchers in computational social science and organisational theory. We further expect the resulting corpus of data to be of considerable interest for the network science and social network analysis community, which have recently developed techniques that incorporate the chronological ordering of interactions in temporal networks and data capturing paths (Newman 2018; Holme 2015; Scholtes 2017b; Lambiotte et al. 2019). We thus hope that the tool and analyses presented in our work will serve the growing community of interdisciplinary researchers working at the intersection of data science, (social) network analysis, computational social science and empirical software engineering.

Finally, the extraction of line-editing paths added in this extended version of our work facilitates the development of novel recommendation tools for practitioners. E.g. models allowing to predict lines that are likely to contain bugs or need additional attention can be trained based on the editing history of both the line as well as similar lines the present as well as other similar projects. This information could further be used to recommend a set of developers best suited to fix bugs or make changes in particular parts of the code. Further, algorithms predicting the refactoring of code can be trained on data, since git2net extracts information on when lines are copied both within and between files using git blame’s -C option.

## Threats to Validity

In the following, we discuss treats to the validity of our work. We split this discussion into two parts. First, we discuss the threats to validity of git2net. We separately focus on the large-scale analysis of coordination overhead in software teams presented in Section 5.

### Threats to Validity for git2net

All threats to validity for git2net are related to the interpretation of the extracted measures. With git2net, we primarily focus on co-editing relationships between different developers. We do this by linking developers consecutively editing the same line of code in a project. Interpreting the resulting co-editing networks as communication or collaboration networks might be invalid due to several reasons. On the one hand, co-editing networks can underestimate collaboration present in the development team. E.g. adding additional lines to a function also requires the developer to understand the remainder of the function. However, links between the corresponding developers are never extracted by git2net. Note, though, that if it is known that and to which extent these links should be present for a given project they can be obtained from the mined database, as git2net records line numbers for all lines. On the other hand, co-editing networks can also overestimate collaboration. This occurs, e.g., in situations where only variable names rather than the structure of the code is changed and hence no coordination between subsequent developers is required. This can be addressed by using code complexity measures such as the Halstead effort (Halstead et al. 1977) of a change as weights in the co-editing network. Alternatively, git blame could be replaced by the recently developed cregit (German et al. 2019) that provides blame information at a token level and thus allows to distinguish different edit types. We aim to explore both options in future versions of git2net.

A second threat to validity to all studies using the cyclomatic complexity originates from git2net extracting the cyclomatic complexity at the level of files, whereas all other analyses are done at line or character level. Due to the definition of cyclomatic complexity it is not possible to fully remove this discrepancy in granularity. We aim to mitigate this issue in a future version of git2net by (i) extracting the cyclomatic complexity of individual functions within a file and (ii) reporting the change in cyclomatic complexity made with each commit for all files.

Thirdly, we note that the accuracy of all results produced by git2net is influenced by the options used with git blame. As most options have both benefits and drawbacks we leave it to users of git2net to select the most appropriate options for their specific use cases. To facilitate this selection, we have detailed the most important options as well as a motivation for our choices for default values in Section 3.1.

Finally, we address some threats to validity to our proposed text entropy measure as well as the block-based extraction and analysis approach. Both approaches were developed specifically to be programming language agnostic. This has the benefit of making them applicable to any file in a repository. However, it comes with the drawback that no code-specific information can be used in their computation. Analysing the code itself could, e.g., allow the block-based approach to split a given block into sub-blocks if they belong to different functions, or combine different blocks if they are referring to the same function. Aside from the limitation to a specific set of programming languages, a second important drawback of parsing the code are the significantly increased computational costs for the analysis. We believe the benefits of a programming language agnostic approach outweigh these drawbacks for most applications, however users of git2net should be aware and potentially adapt their approach in case their application demands it.

### Threats to Validity for the Large-Scale Coordination Study

Next, we address the threats to validity of our large-scale of coordination in Open Source Software development teams.

#### Construct validity

First, we refer to potential threats to validity with regard to our measures. For our study, we measure developer productivity with respect to edits made to previously developed code. Defining productivity as Levenshtein edit distance per commit, we use the time between two consecutive commits by a developer as upper bound for the time a developer spent on these changes. While we argue that this is the best proxy available in our data, inter-commit times also include other developer activities such as the simple addition of code (without modifying existing code), deletion of existing code, eating, sleeping, etc. By aggregating over a large number of commits, these activities should, however, have little impact on our conclusions. Further, we found cases, particularly for linux, where the the assumption that inter-commit times represent an upper boundary for the time in which the corresponding edits were made was violated. We addressed this issue by aggregating commits to contributions (cf. Section S1). We therefore argue that our measure is a meaningful proxy of developer productivity within the context of our study.

Defining productivity as the amount of code modified in a time interval, we note that our results cannot be used to make claims about the quality of the produced code (and hence team performance).

#### Internal validity

Due to the large computational requirements of git blame, we could not extract some commits (mostly merges) from the respective databases (cf. Section 5.3). While we do not expect that the small fraction of commits excluded from our analysis has a considerable impact on the final results, we cannot conclusively rule out this possibility.

Further, during the data cleaning process, we have selected thresholds for the aggregation of commits to contributions as well as the subsequent removal of outlier observations present due to automated changes and search/replace operations. To address this potential issue, we have performed robustness checks on our results in which we test the impact of these parameters (cf. Section S5). This analysis shows that none of our conclusion changes when varying the parameters. In all cases, the inclusion of *own* significantly improves the model based on AIC as ME+ is selected as optimal model in all cases. Further for larger projects the significance of our findings does not change. However, due to the smaller size of the data for igraph and bitcoin, we find that an excessive aggregation of commits might lead to an increase in the *p*-value. In future works, we will develop more granular filters to allow project specific data cleaning.

A further threat to validity originates from omitted-variable bias, i.e. unobserved features that might influence developer productivity. Examples could, e.g., be approaching deadlines (Costello 1984), developer emotions (Garcia et al. 2013), internal team challenges such as changes in the team structure, working environments, or resource constraints (Alliger et al. 2015) that were not considered in this study. Similarly, we did not further explore potential interactions between the considered controls.

Finally, for any study based on git author disambiguation presents a threat to validity as it adds in both the data collection as well as all subsequent analyses. The disambiguation approach used in this analysis is based on simple heuristics and is therefore able to scale well to the large data sets used in our study. However, since conducting our study, new advanced disambiguation algorithms and data sets have been made available which should be considered for future studies (Amreen et al. 2020; Fry et al. 2020).

#### External validity

Finally, we want to discuss the threats to the generalisability of our results. The six projects involved in our study represented a variety of project sizes as well as topics. We find strong evidence for our hypothesis in all six projects. Despite this finding, we cannot confidently rule out that other sets of projects could yield different findings. In future work, we will aim to address this by considering additional projects selected based on predefined sets of criteria. We specifically intend to repeat our analysis on the 58 projects used in (Scholtes et al. 2016), testing whether the project-specific differences in the magnitude of the Ringelmann effect can be explained by the variations in the impact of co-editing patterns on developer productivity discovered in the present work.

## Conclusion

Over the past two decades, the analysis of co-authorship, co-commit, or co-editing networks in software development teams has experienced huge interest from the empirical software engineering and repository mining community. Exemplary studies have shown that the analysis of such collaboration networks helps to assess the time-evolving social structure of teams (Scholtes et al. 2016; Madey et al. 2002), predict software defects (Meneely et al. 2008), categorise developer roles (Pohl and Diehl 2008), identify communities (Joblin et al. 2015), or study knowledge spillover across individuals, teams, and projects (Von Krogh and Von Hippel 2006; Vijayaraghavan et al. 2015; Huang and Liu 2005; Cohen and Consens 2018). Most of these studies have employed definitions of co-authorship networks which assume that developers are linked if they edited a common file, module, or binary. However, such coarse-grained definitions have been shown to neglect information on the microscopic patterns of collaborations contained in the time-ordered sequence of lines of code edited by developers (Joblin et al. 2015; Scholtes et al. 2016).

To facilitate data-driven studies of developer networks that take advantage of this detailed information, we introduce git2net, a python package for the mining of fine-grained and time-stamped collaboration networks from large git repositories. Going beyond previous works, we adopt text mining techniques to assess (a) the development effort of an edit in terms of the Levenshtein distance between the version before and after the commit, and (b) the entropy of file modifications, which can be used to filter out changes in text-encoded binary data. Thanks to a parallel processing model our tool exhibits a linear speed up for an increasing number of processing cores. This makes git2net suitable to analyse git repositories with hundreds of thousands of commits and millions of lines of code.

Apart from a description of our tool, we demonstrate that git2net simplifies the construction and analysis of dynamic developer collaboration networks and co-editing behaviour in real data on Open Source and proprietary software projects. Extending the analysis presented by Joblin et al. (2015), in Section 4.2 we perform a comparative study of a file- vs. line-based construction of co-editing networks. In Section 4.4 we further demonstrate that the information extracted by our tool can be used to generate a time-resolved breakdown of developer effort into (a) the revision of code authored by the developer her- or himself vs. (b) the revision of code written by other team members.

Building on this information, we perform a large-scale study on coordination overhead as a driving factor of the Ringelmann effect in six Open Source Software projects. Using fine-grained co-editing networks extracted from repository data that covers more than 1.2 million commits from more than 25,000 developers, we find strong evidence for the hypothesis that the editing of code previously written by others negatively impacts the individual productivity of developers. We further find that the negative impact of coordination on developer productivity exhibits strong variation across different projects. With this, we provide a potential additional explanation for the variation in the magnitude of the Ringelmann effect across projects found in prior studies.

## Tool Availability, Archival, and Reproducibility

The tool presented in this work is available as Open Source software package on GitHub^6^. git2net is further available via the python package index pypi, enabling users to simply install and update it via the package management tool pip. To support the reproducibility of our work, we have permanently archived the version of our tool that was used to obtain the results presented in this paper on the open-access repository zenodo.org (Gote et al. 2019a).

git2net comes with unit tests and a comprehensive in-line documentation. To support users in developing their first analysis, we further provide access to interactive jupyter notebooks, including a detailed tutorial which allow to reproduce our approach.

## Electronic supplementary material

Below is the link to the electronic supplementary material.

(PDF 389 KB)

## Footnotes

1 https://github.com/gotec/git2net

2 The results can be reproduced by running git2net.mine_git_repo(‘igraph’, ‘igraph.db’, num_processes=N) for different *N* from within the python API of git2net. The versions of git2net, the igraph repository, as well as all raw data to produce the figures shown in this manuscript are available on zenodo.org (Gote et al. 2019a).

3 Intel®; Core™ i9 7960X, 16C/32T, 2.80GHz base, 4.2GHz boost

4 c.f. commit *eaaface92ee81f30a6ac66fe7acbcc42c00dc450* in gentoo

5 Results for the other projects are shown in Section S3.

6 https://github.com/gotec/git2net
