# Supplementary material for: Analysing Time-Stamped Co-Editing Networks in Software Development Teams using git2net
Source: Empir Softw Eng. 2021 May 26;26(4):75. doi: 10.1007/s10664-020-09928-2 (PMC8550015; doi:10.1007/s10664-020-09928-2)
Supplement: Supplementary file 1 — (PDF 389 KB) [file 10664_2020_9928_MOESM1_ESM.pdf]

**Empirical Software Engineering manuscript No. EMSE-D-19-00234**

# Supplementary Material: Analysing Time-Stamped Co-Editing Networks in Software Development Teams using git2net

**Christoph Gote · Ingo Scholtes · Frank Schweitzer**

Accepted: 17 December 2020

In the following we provide additional information and supporting results for the large scale analysis of coordination overhead in software teams presented in section 5 of the main manuscript.

First, in section S1 we describe in detail the data cleaning process underlying our results and provide additional information on the resulting data in section S2. Subsequently, we show feature correlations before and after feature selection for `igraph`, `bitcoin`, `libav`, `FFmpeg`, and `gentoo` in section S3. Further, we show all results from the model selection step in section S4. Finally, we present additional results supporting the robustness of our findings with regard to the parameters set during the data cleaning step are in section S5.

Christoph Gote
Chair of Systems Design
ETH Zurich
Zurich, Switzerland
E-mail: cgote@ethz.ch

Ingo Scholtes
Data Analytics Group
Bergische Universität Wuppertal
Wuppertal, Germany
E-mail: scholtes@uni-wuppertal.de

Frank Schweitzer
Chair of Systems Design
ETH Zurich
Zurich, Switzerland
E-mail: fschweitzer@ethz.ch

## S1 Data cleaning

As discussed in section 5.2 of the main manuscript, to test our research hypothesis, only edits in which existing code was modified are relevant. Therefore, any commits that are not labelled as "replacement" by `git2net` are dropped. Any edits that originated from or resulted in an empty line are dropped for the same reason. Additionally, we do not consider any edits to files for which no cyclomatic complexity can be computed. These are generally data files, images, etc. which are not part of this analysis. The exact number of disregarded edits per project are shown in Table S1.

While cleaning the data, we discovered a large number of commits with inter-commit times of 0 and 1 seconds, particularly for `linux`. Further analysis revealed that developers often make multiple consecutive commits after working on a section of code. In doing so, individual commit messages can be assigned to different sets of edits, facilitating the tracking of changes in the project. This behaviour invalidates our assumption that the inter-commit time represents an upper boundary of the time a developer spent on the edits is violated. As illustrated in Figure S1 we thus aggregate commits with inter-commit times of less than a given threshold $\Delta$ to a single code contribution. While the threshold needs to be sufficiently high to avoid the cases mentioned above, setting it too high will merge commits that belong to adjacent contributions. After discussions with professional software developers, we aggregated consecutive commits with inter-commit times of less that $\Delta = 5$ minutes[1]. Subsequently, we perform all analyses at the level of (aggregated) code contributions rather than commits.

The `GitHub` repository of `gentoo` only exists since August 2015, whereas development started as early as 1999. When creating the `git` repository, an initial commit was made that includes the entire history of the project until this point. To not falsely attribute all previous development efforts to the author making this first commit, we drop all edits to lines initially added with the first commit. This amounts to almost 25% of the remaining edits in the database.

The distribution of developer productivity reveals a small number of outliers with very large values. A manual inspection showed that these are mostly due to automated changes of code style, or search and replace operations[2]. We argue that such commits are not representative of typical software development and thus consider them as outliers. To not bias our analysis, we removed them from the dataset by excluding the top $\varepsilon$ quantile of contributions with respect to productivity. Similar to the aggregation time window, the removal threshold cannot be set too low, but setting it too high will also result in the removal of the

[1] We highlight that our results are robust with regard to different parameters $\Delta$. Results for $\Delta$ of 1 and 10 minutes are shown in the supplementary material.

[2] cf. commit *4be44fcd3bf648b782f4460fd06dfae6c42ded4b* in `linux` or commit *eaaface92ee81f30a6ac66fe7acbcc42c00dc450* in `gentoo`

Table S1: Overview of data collection and cleaning process. Only commits with less than 1000 modified files were originally mined. Edits were dropped due to not being replacements or not relating to code.

| | **igraph** | **bitcoin** | **libav** | **FFmpeg** | **gentoo** | **linux** |
|---|---|---|---|---|---|---|
| # of authors | 36 | 803 | 965 | 1,785 | 964 | 20,581 |
| # of commits | 5,919 | 21,196 | 45,232 | 94,942 | 265,453 | 855,283 |
| # mined | 5,885 | 20,545 | 45,232 | 90,197 | 264,559 | 814,535 |
| % mined | 99.43 | 96.93 | 100.00 | 95.00 | 99.66 | 95.24 |
| # of replacements | 85,650 | 338,733 | 551,468 | 835,141 | 376,102 | 6,985,866 |
| # dropped | 13,877 | 70,060 | 23,045 | 31,319 | 3,464 | 227,007 |
| % dropped | 13.94 | 17.14 | 4.01 | 3.61 | 0.91 | 3.15 |

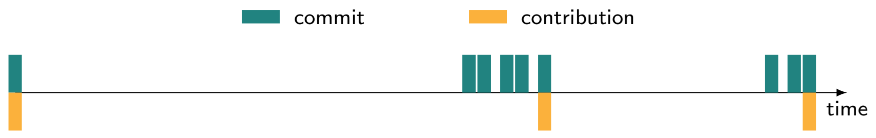


Fig. S1: Aggregation of commits to contributions.

most productive contributions in the respective project. After discussion with professional developers, we decided to remove the top $\varepsilon = 5\%$ contributions with regard to developer productivity from the dataset[3].

The `git` protocol allows developers to use any name and email address when making commits. Hence it frequently occurs that the same developer appears with differently spelt names (e.g. in the case of names with special characters) or different email addresses (e.g. work and personal emails) in the same repository (Bird et al., 2009; German, 2004). This is an essential problem as it adds noise to both the data collection and any subsequent analyses. Unfortunately, it is also a challenging problem, particularly when dealing with large-scale data. Aiming to correctly disambiguate authors, we compared two heuristic-based approaches. For the first approach, we matched authors based on the author email recorded in `git`. The second approach performs a matching based on the recorded author names after removing capitalisation and special characters. Upon comparison with a manually created ground-truth for the `igraph` data set, we found that the second approach yielded better performance. Therefore, this approach was used for all projects.

## S2 Data description

Figure S2 provides an overview of the amount and types of edits made in the projects included in our large-scale analysis of coordination overhead in software teams based on a line-based extraction of edits. The first two columns show edit counts as well as relative edit counts for a moving window of 295 days. The window size was selected based on the finding of Scholtes et al. (2016) that after 295 days of inactivity, the probability of a subsequent commit of an Open Source Software developer is less than 10% and hence the developer should no longer be considered as member of the development team. We find that (with values ranging between 60 and 90%) additions make up most of the largest parts of all edits across projects. In contrast almost no code is deleted without being replaced as can be seen from the very low amount of deletions. Code replacements, where an existing line is edited, make up around 20% of the data. As we aim to study coordination overhead, code replacements are the main focus of our analysis as these allow us to directly link the consecutive developers editing the same line with a co-editing relationship.

The third column shows the count of replacements for which code by other authors is edited as fraction of the total number of code replacements. Colours show the development of both team size and communication requirements over time. The dashed line shows a linear model of the form $y = \alpha x + \beta$ fitted to the data. We find that the slope is positive and significant for all projects indicating an increase in coordination requirements for larger teams. These results confirm the findings made in section 5.1 in the main manuscript for Levenshtein edit distance also for count data.

## S3 Feature correlations

In this section, we report the feature correlations used for the feature selection in section 5.2 of the main manuscript. There, a description of the all features is provided in Table 2. For the feature correlations for the `linux` kernel development project please refer to Figures 13 and 14 in the main manuscript.

[3] The results are robust with regard to the removal threshold. Results for $\varepsilon = 1\%$ and 10% are shown in the supplementary material (section S5).

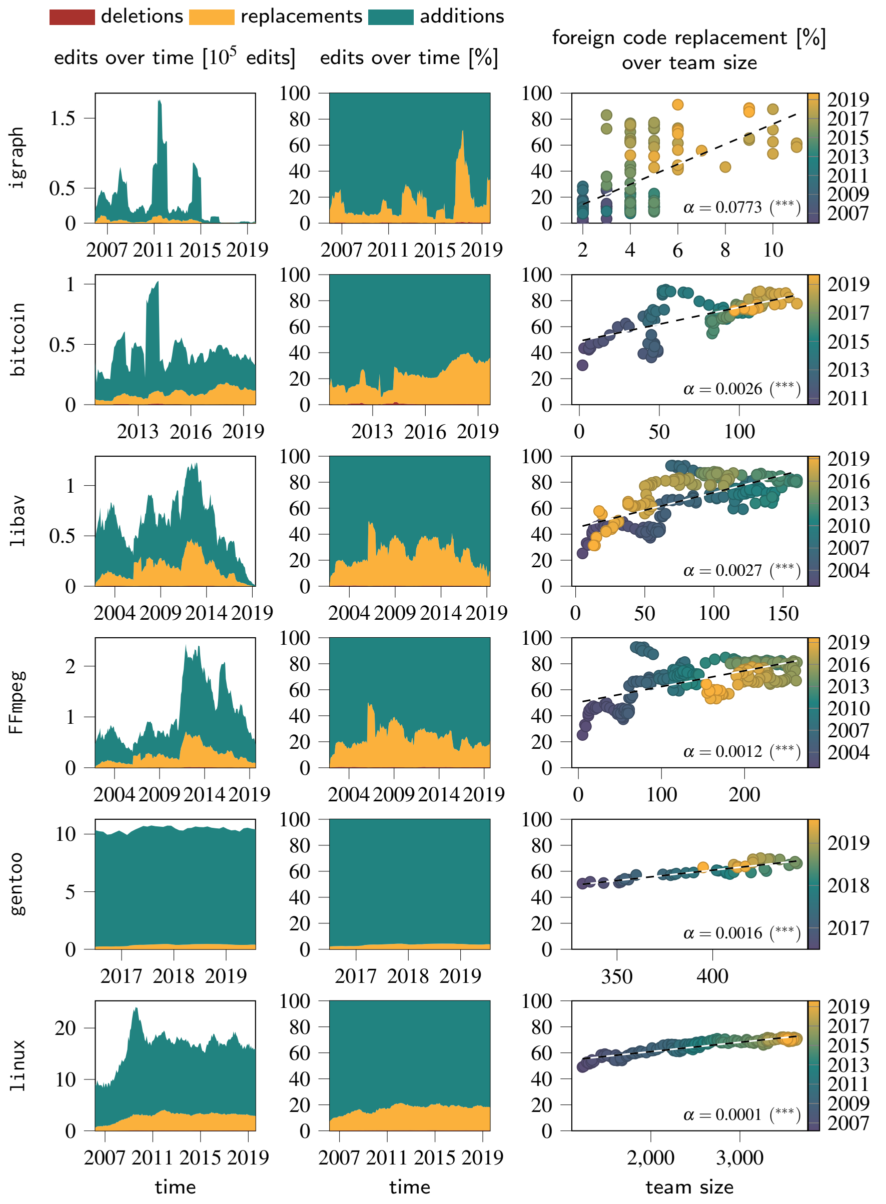


Fig. S2: Summary statistics for all edits made in the repositories analysed in section 5 of the main manusc based on a rolling window analysis with a window size of 295 days and a time increment of four weeks. The first column shows the total count of lines edits made within the time window. Colours show the subdivision of the edits into deletions, replacements, and additions. The second column shows the proportion of the different edit types over time. Deletions only account for a very small fraction of edits and replacements amount to around 20% of the edits made. The third column shows the fraction of foreign code replacement for different team sizes based on counts. Colours show the development over time. The parameter $\alpha$ represents the slope of a linear $y = \alpha x + \beta$ for the the plots. The asterisks indicate $p < 0.05$ (*), $p < 0.01$ (**), and $p < 0.001$ (***) for the slope being positive. Results confirm the findings of section 5 of the main manuscript for count data.

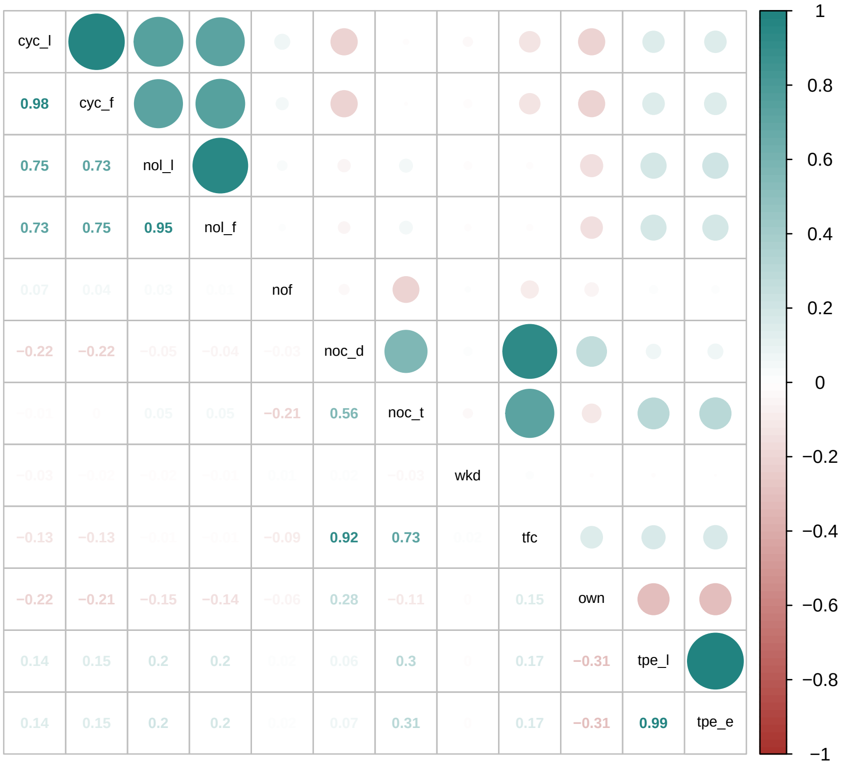


Fig. S3: Spearman's rank-order correlations for `igraph` before feature selection.

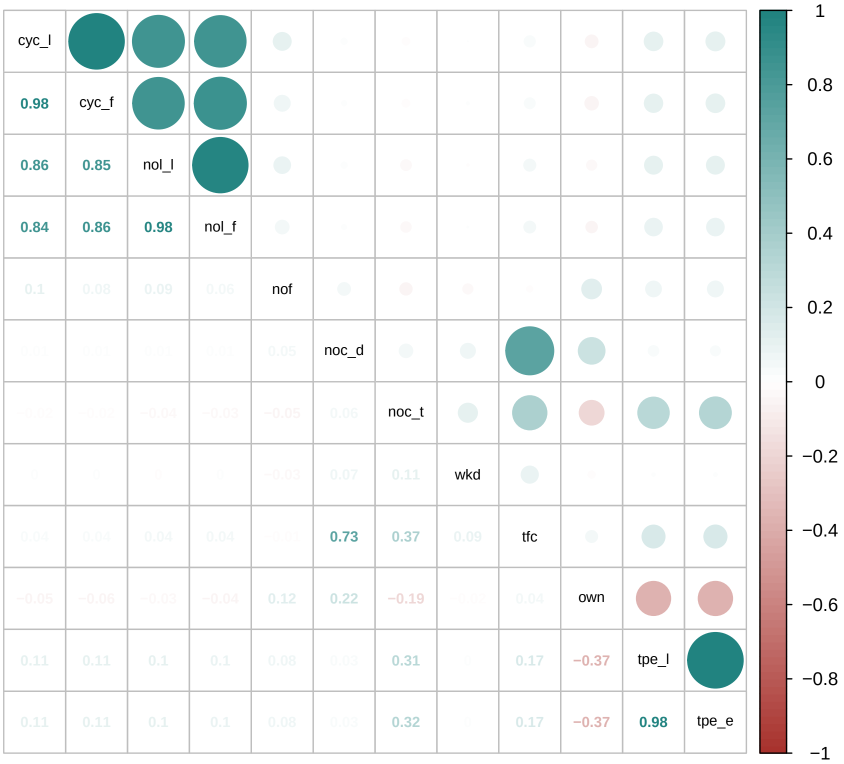


Fig. S4: Spearman's rank-order correlations for `bitcoin` before feature selection.

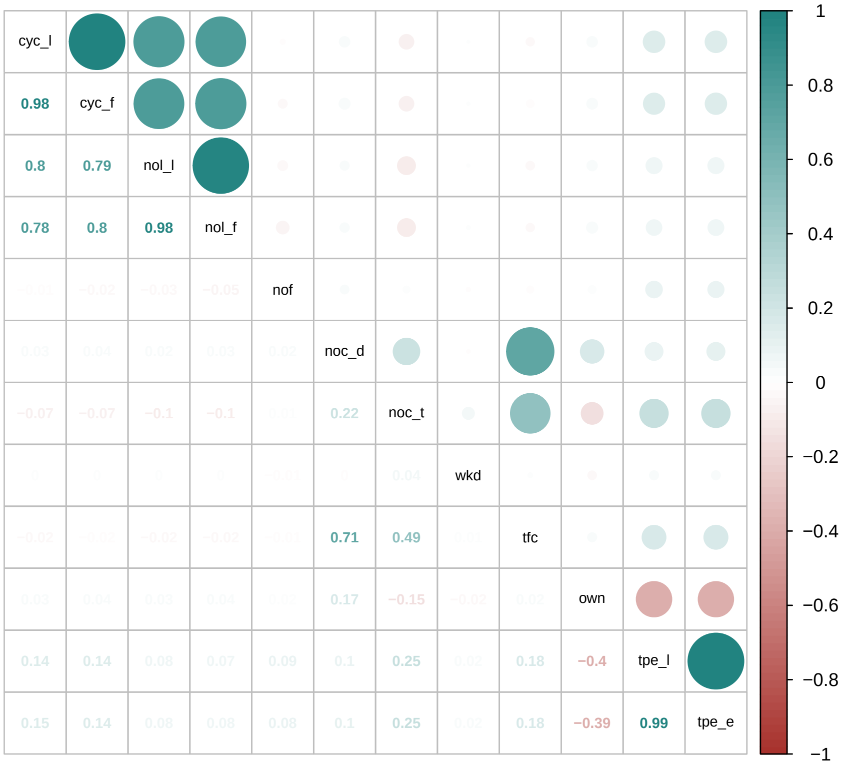

Fig. S5: Spearman's rank-order correlations for `libav` before feature selection.

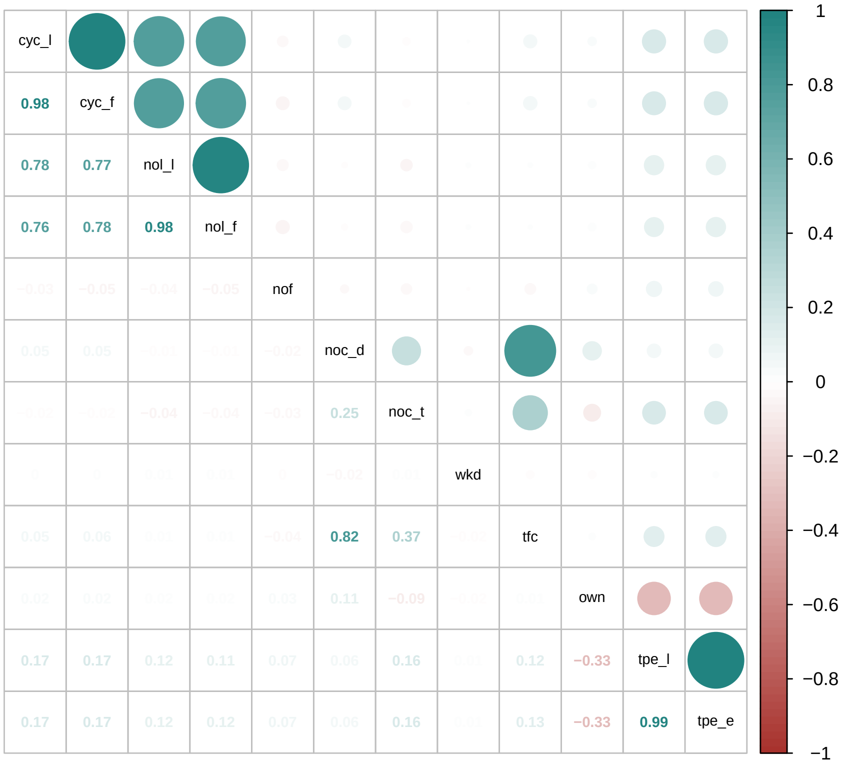

Fig. S6: Spearman's rank-order correlations for `FFmpeg` before feature selection.

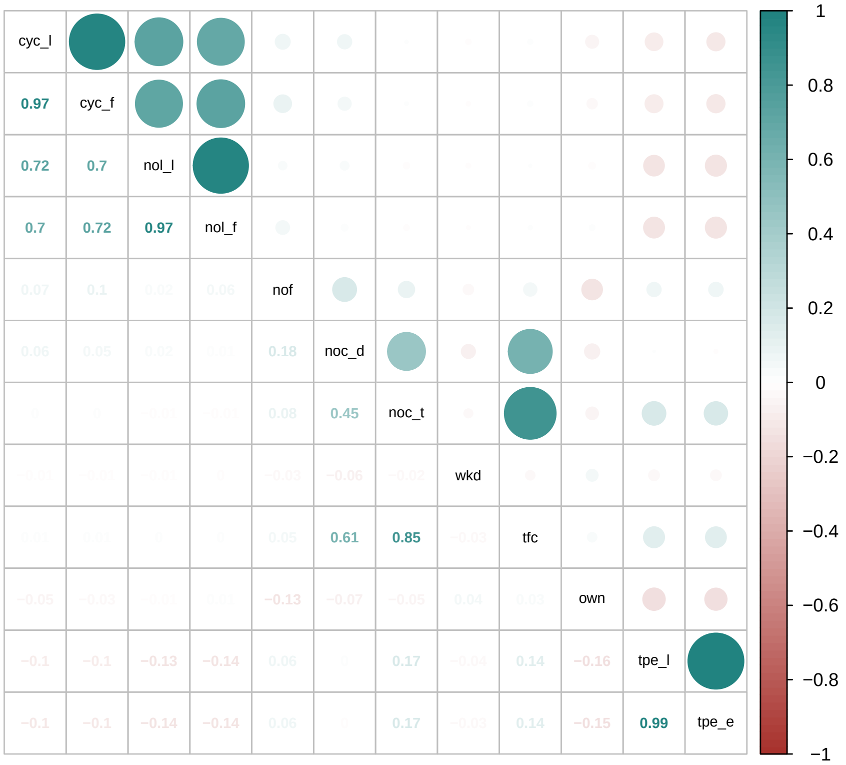


Fig. S7: Spearman's rank-order correlations for `gentoo` before feature selection.

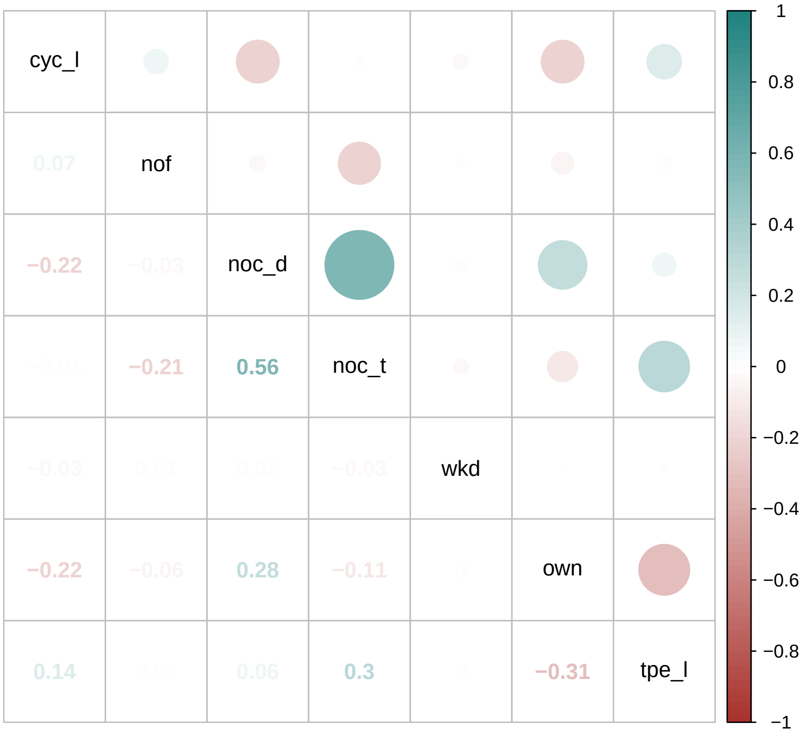


Fig. S8: Spearman's rank-order correlations for `igraph` after feature selection.

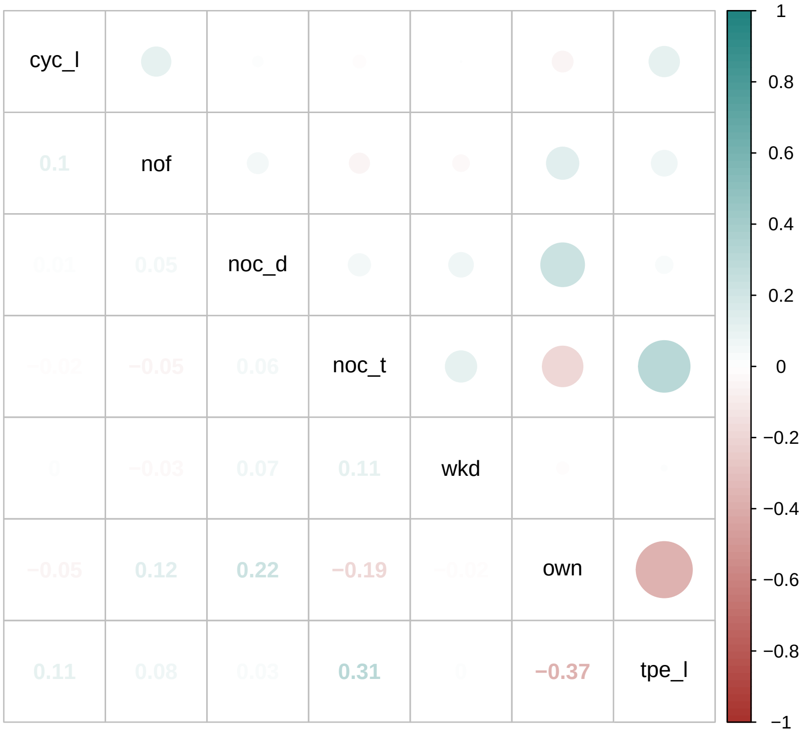


Fig. S9: Spearman's rank-order correlations for `bitcoin` after feature selection.

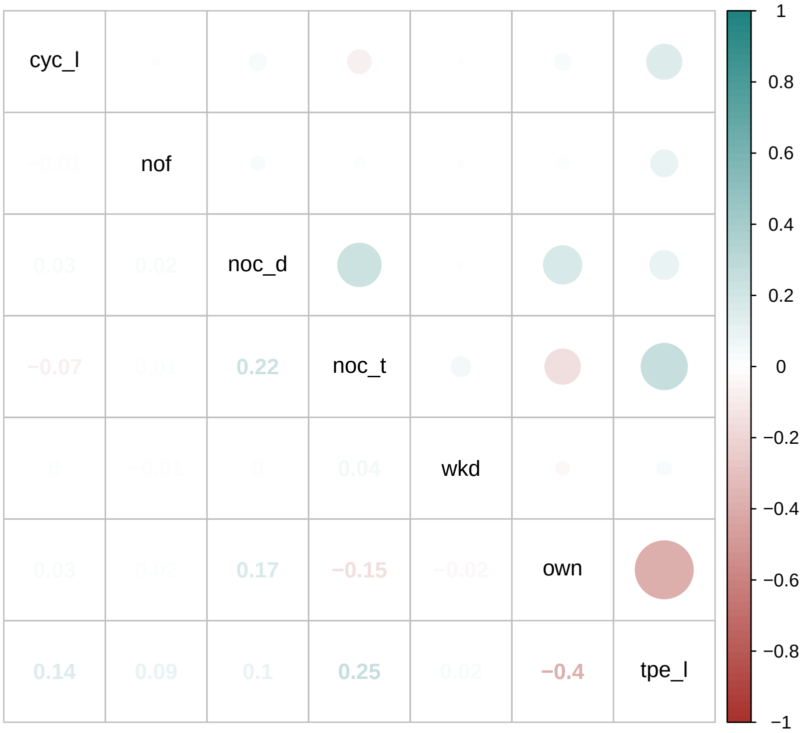


Fig. S10: Spearman's rank-order correlations for `libav` after feature selection.

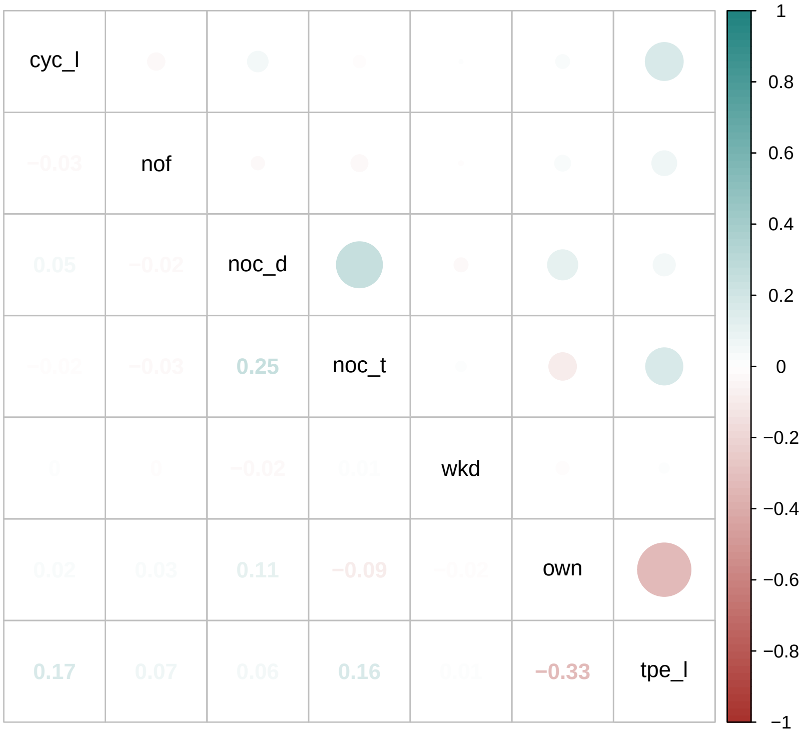


Fig. S11: Spearman's rank-order correlations for `FFmpeg` after feature selection.

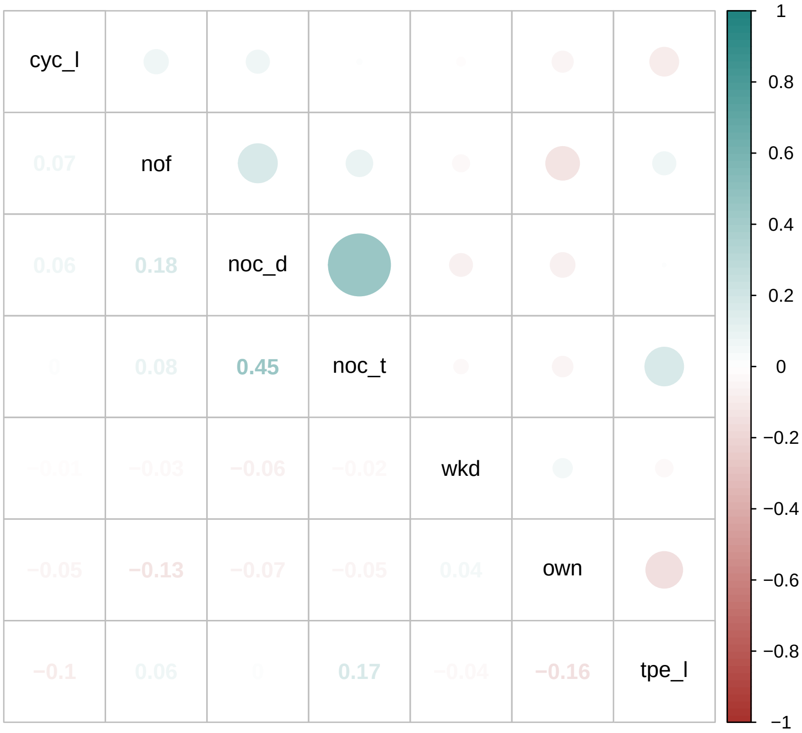


Fig. S12: Spearman's rank-order correlations for `gentoo` after feature selection.

## S4 Model selection

In this section, we report the AIC as well as Chi-square test based model selection results finding that ME+ is the most suitable model to describe the productivity in all considered projects. The three candidate models models are defined in Table 4 of the main manuscript. For the model selection results for the `linux` kernel development project please refer to Table 5.

Table S2: AIC as well as Chi-squared test for the three candidate models for `igraph`.

| igraph | Df | AIC | Chisq | Chi Df | Pr(>Chisq) |
|---|---|---|---|---|---|
| LM | 9 | 16.75 | — | — | — |
| ME- | 11 | 11.39 | 9.36 | 2.0 | 0.01 |
| **ME+** | 12 | 0.00 | 13.39 | 1.0 | 0.00 |

Table S3: AIC as well as Chi-squared test for the three candidate models for `bitcoin`.

| bitcoin | Df | AIC | Chisq | Chi Df | Pr(>Chisq) |
|---|---|---|---|---|---|
| LM | 9 | 69.40 | — | — | — |
| ME- | 11 | 8.69 | 64.71 | 2.0 | 0.0 |
| **ME+** | 12 | 0.00 | 10.69 | 1.0 | 0.0 |

Table S4: AIC as well as Chi-squared test for the three candidate models for `libav`.

| libav | Df | AIC | Chisq | Chi Df | Pr(>Chisq) |
|---|---|---|---|---|---|
| LM | 9 | 362.64 | — | — | — |
| ME- | 11 | 38.31 | 328.33 | 2.0 | 0.0 |
| **ME+** | 12 | 0.00 | 40.31 | 1.0 | 0.0 |

Table S5: AIC as well as Chi-squared test for the three candidate models for `FFmpeg`.

| FFmpeg | Df | AIC | Chisq | Chi Df | Pr(>Chisq) |
|---|---|---|---|---|---|
| LM | 9 | 787.62 | — | — | — |
| ME- | 11 | 13.95 | 777.67 | 2.0 | 0.0 |
| **ME+** | 12 | 0.00 | 15.95 | 1.0 | 0.0 |

Table S6: AIC as well as Chi-squared test for the three candidate models for `gentoo`.

| gentoo | Df | AIC | Chisq | Chi Df | Pr(>Chisq) |
|---|---|---|---|---|---|
| LM | 9 | 1364.22 | — | — | — |
| ME- | 11 | 36.72 | 1331.50 | 2.0 | 0.0 |
| ¡ **ME+** | 12 | 0.00 | 38.72 | 1.0 | 0.0 |

## S5 Results for alternative cleaning parameters

In this section, we report the regression results of our large-scale analysis of coordination overhead in software teams for alternative data cleaning parameters. The results show that our findings are robust against

the parameters for commit aggregation ($\Delta$) and outlier removal ($\varepsilon$) selected during the data cleaning process described in section S1. For a full description of the results with the selected parameters please refer to Table 6 and section 5.4 of the main manuscript.

Table S7: Regression results of ME+ for all projects in the case study for $\Delta = 1$ minute and $\varepsilon = 5\%$. The asterisks indicate $p < 0.05$ (*), $p < 0.01$ (**), and $p < 0.001$ (***).

| | igraph | bitcoin | libav | FFmpeg | gentoo | linux |
|---|---|---|---|---|---|---|
| $own$ | -20.72 | 22.11** | 33.11*** | 11.95*** | 4.43* | 11.04*** |
| (IC) | 178.63* | 63.24*** | 115.12*** | 99.37*** | 85.98*** | 91.27*** |
| $cyc_l$ | 0.1* | 0.01 | 0.0 | -0.0 | -1.16*** | 0.0 |
| $noc_t$ | 2.16 | -0.78 | -2.11*** | -0.59*** | -0.11*** | -0.09*** |
| $nof$ | 3.68** | 2.04*** | 1.22*** | 1.6*** | 4.17*** | 3.09*** |
| $tpe_l$ | -2.44 | 4.1 | 11.48*** | 4.91*** | 47.65*** | 4.35*** |
| $noc_d$ | -0.15 | 7.3* | 5.39** | 3.8*** | 1.52*** | 5.18*** |
| $wkd$ | -12.06 | 4.93 | -9.56* | -6.94* | -10.26*** | -13.59*** |

Table S8: Regression results of ME+ for all projects in the case study for $\Delta = 10$ minutes and $\varepsilon = 5\%$. The asterisks indicate $p < 0.05$ (*), $p < 0.01$ (**), and $p < 0.001$ (***).

| | igraph | bitcoin | libav | FFmpeg | gentoo | linux |
|---|---|---|---|---|---|---|
| $own$ | 25.28** | 3.9 | 9.28*** | 4.17*** | 5.02*** | 3.25*** |
| (IC) | 15.47 | 26.67*** | 28.53*** | 24.28*** | 18.7*** | 10.65*** |
| $cyc_l$ | 0.04** | 0.01* | -0.0 | -0.0 | -0.2*** | 0.0*** |
| $noc_t$ | 2.99 | -0.39 | -0.45*** | -0.12*** | -0.04*** | -0.01*** |
| $nof$ | 1.98*** | 0.71*** | 1.12*** | 1.34*** | 0.86*** | 1.07*** |
| $tpe_l$ | -0.01 | -0.75 | 3.05*** | 1.53*** | 7.6*** | 0.57*** |
| $noc_d$ | -0.09 | 3.88· | 1.69*** | 0.97*** | 0.2*** | 1.03*** |
| $wkd$ | 2.81 | 5.94· | -4.24** | -2.26* | -1.98** | -0.74* |

Table S9: Regression results of ME+ for all projects in the case study for $\Delta = 5$ minutes and $\varepsilon = 1\%$. The asterisks indicate $p < 0.05$ (*), $p < 0.01$ (**), and $p < 0.001$ (***).

| | igraph | bitcoin | libav | FFmpeg | gentoo | linux |
|---|---|---|---|---|---|---|
| $own$ | -23.15 | 22.41 | 30.08*** | 10.93* | 18.39*** | 12.57*** |
| (IC) | 111.24** | 45.09* | 89.25*** | 82.3*** | 83.09*** | 62.14*** |
| $cyc_l$ | 0.09* | 0.06** | -0.0 | -0.01· | -1.28*** | 0.01** |
| $noc_t$ | -4.07 | -0.1 | -1.36** | -0.37** | -0.12*** | -0.08*** |
| $nof$ | 6.01*** | 4.35*** | 2.79*** | 3.54*** | 2.52*** | 6.39*** |
| $tpe_l$ | -3.91 | 9.95* | 14.54*** | 5.48*** | 32.17*** | 4.67*** |
| $noc_d$ | 2.36 | 10.6*** | 3.16* | 2.59*** | 0.67* | 4.7*** |
| $wkd$ | 9.05 | 6.74 | -14.2* | -7.4· | -9.55** | -12.5*** |

Table S10: Regression results of ME+ for all projects in the case study for $\Delta = 5$ minutes and $\varepsilon = 10\%$. The asterisks indicate $p < 0.05$ (*), $p < 0.01$ (**), and $p < 0.001$ (***).

| | igraph | bitcoin | libav | FFmpeg | gentoo | linux |
|---|---|---|---|---|---|---|
| *own* | 12.36$^{*}$ | 3.71· | 8.47$^{***}$ | 3.58$^{***}$ | 2.61$^{***}$ | 1.71$^{***}$ |
| (IC) | 25.66$^{***}$ | 22.49$^{***}$ | 27.07$^{***}$ | 21.05$^{***}$ | 19.19$^{***}$ | 7.67$^{***}$ |
| $cyc_l$ | 0.0 | 0.0 | -0.0 | -0.0 | -0.19$^{***}$ | 0.0$^{*}$ |
| $noc_t$ | 0.86 | -0.62$^{**}$ | -0.49$^{***}$ | -0.12$^{***}$ | -0.03$^{***}$ | -0.01$^{***}$ |
| $nof$ | 1.08$^{***}$ | 0.37$^{***}$ | 0.77$^{***}$ | 0.91$^{***}$ | 0.7$^{***}$ | 0.62$^{***}$ |
| $tpe_l$ | -0.22 | 0.65 | 2.5$^{***}$ | 1.2$^{***}$ | 6.7$^{***}$ | 0.34$^{***}$ |
| $noc_d$ | 28.26 | 2.45$^{**}$ | 1.79$^{***}$ | 1.08$^{***}$ | 0.25$^{***}$ | 0.76$^{***}$ |
| $wkd$ | 1.83 | 3.78· | -3.84$^{**}$ | -1.96$^{**}$ | -1.84$^{***}$ | -0.33· |

## References


Bird C, Rigby PC, Barr ET, Hamilton DJ, German DM, Devanbu P (2009) The promises and perils of mining git. In: 2009 6th IEEE International Working Conference on Mining Software Repositories, IEEE, pp 1–10

German DM (2004) Mining CVS repositories, the softChange experience. In: MSR, Citeseer, vol 4, pp 17–21

Scholtes I, Mavrodiev P, Schweitzer F (2016) From Aristotle to Ringelmann: A large-scale analysis of team productivity and coordination in Open Source Software projects. Empirical Software Engineering 21(2):642–683
